# Di-μ_3_-chlorido-1:2:3κ^3^*Cl*;2:3:4κ^3^*Cl*-di-μ_2_-chlorido-1:2κ^2^*Cl*;3:4κ^2^*Cl*-tetra­kis­[(4-amino-1,5-dimethyl-2-phenyl-2,3-di­hydro-1*H*-pyrazol-3-one-κ^2^*N*^4^,*O*)chlorido­cadmium(II)] 1.7-hydrate: a new six-coordinate geometry index, τ_6_

**DOI:** 10.1107/S2056989025003123

**Published:** 2025-04-11

**Authors:** Helen Stoeckli-Evans, M. G. Shankar, R. Kumaravel, A. Subashini, T. Sabari Girisun, K. Ramamurthi, Monika Kučeráková, Michal Dušek, Aurélien Crochet

**Affiliations:** a Institute of Physics, University of Neuchâtel, Rue Emile-Argand 11, CH-2000 Neuchâtel, Switzerland; b PG and Research Department of Physics Srimad Andavan Arts and Science College (Autonomous) Affiliated to Bharathidasan University, Tiruchirappalli 620005 Tamilnadu India; c Department of Physics, Annapoorana Engineering College (Autonomous), Salem 636308, Tamilnadu, India; d Nanophotonics Laboratory Department of Physics Bharathidasan University, Tiruchirappalli 620024 Tamilnadu India; e Crystal Growth and Thin Film Laboratory Department of Physics Bharathidasan University, Tiruchirappalli 620024 Tamilnadu India; f Institute of Physics ASCR Na Slovance 2 182 21 Praha 8 Czech Republic; g Chemistry Department, University of Fribourg, Chemin du Musée 9, CH-1700 Fribourg, Switzerland; University of Aberdeen, United Kingdom

**Keywords:** crystal structure, 4-amino­anti­pyrine, cadmium(II), tetra­nuclear, six-coordinate geometry index, hy­dro­gen bonding

## Abstract

The title centrosymmetric tetra­nuclear cadmium(II) com­plex of 4-amino­anti­pyrine and chloride ions, [Cd_4_Cl_8_(C_11_H_13_N_3_O)_4_]·1.7H_2_O, was synthesized using methanol as solvent. The two independent Cd^2+^ ions in the asymmetric unit have different geometries; the outer Cd atoms have fivefold CdONCl_3_ coordination spheres, while the inner Cd atoms have sixfold CdONCl_4_ coordination spheres. The com­plex is consolidated by intra­molecular N—H⋯O and N—H⋯Cl hy­dro­gen bonds. In the crystal, a combination of N—H⋯Cl and O_w_—H⋯Cl (w = water) hy­dro­gen bonds link the com­ponents to form chains propagating along the *a*-axis direction. The chains are crosslinked by C—H⋯Cl and C—H⋯O hy­dro­gen bonds to form a three-dimensional structure. A new geometry index, τ_6_, is proposed to qu­anti­tatively estimate the geometry of a sixfold coordinated atom.

## Abstract 2

A new tetra­nuclear cadmium(II) com­plex of 4-amino­anti­pyrine and chloride ions was synthesized using methanol as solvent. The com­plex possesses inversion symmetry with two independent Cd^2+^ ions that have different coordination spheres, one fivefold and the other sixfold. A new geometry index, τ_6_, is propossed to qu­anti­tatively describe the geometry of a sixfold coordinated atom.

## 1. Chemical context

Anti­pyrine derivatives have gained inter­est as model com­pounds for functional materials due to their various properties, including anti­oxidant (Bashkatova *et al.*, 2005 ▸), anti­pu­trefactive (Abd El Rehim *et al.*, 2001 ▸) and optical properties (Collado *et al.*, 2000 ▸; Coolen *et al.*, 1999 ▸). One such analogue is 4-amino-1,5-dimethyl-2-phenyl­pyrazol-3-one, C_11_H_13_N_3_O, known as 4-amino­anti­pyrine (4-AAP). Its crystal structure was first reported by Li *et al.* (2013 ▸), who also analysed its electronic structure and that of a number of its derivatives, including 4-(di­methyl­amino)­anti­pyrine. A low-tem­per­a­ture structural analysis of 4-AAP has been reported by Mnguni & Lemmerer (2015 ▸). The structure features a five-membered lactam ring in the pyrazole unit and a free amino group. Pyrazolone-based ligands exhibit variable com­plexing behaviour and a variety of coordination possibilities to metal centres. Such com­plexes have applications in both chemistry and the pharmaceutical sciences (Raman *et al.*, 2014 ▸). Derivatives of 4-AAP have also emerged as important com­pounds in the fields of biology and medicine (Senthilkumar *et al.*, 2016 ▸). The presence of heteroatoms influences the electron distribution, which in turn enhances its reactivity and chelating properties (Matczak & Domagała, 2017 ▸; Joule & Mills, 2008 ▸). Due to this excellent chelating effect it can form a wide variety of metal com­plexes with almost all transition-metal ions and lanthanides. They have applications in many fields of research, such as sensor development (Banasz & Wałęsa-Chorab, 2019 ▸), renewable energy materials (Zhang *et al.*, 2018 ▸), chemosensors (Selvan *et al.*, 2016 ▸), DNA binding (Sakthivel *et al.*, 2020 ▸), anti­pyretic (Turan-Zitouni *et al.*, 2001 ▸), anti­oxidant (Bashkatova *et al.*, 2005 ▸), anti­cancer (Bose *et al.*, 2005 ▸) and anti-inflammatory agents (Sondhi *et al.*, 1999 ▸).
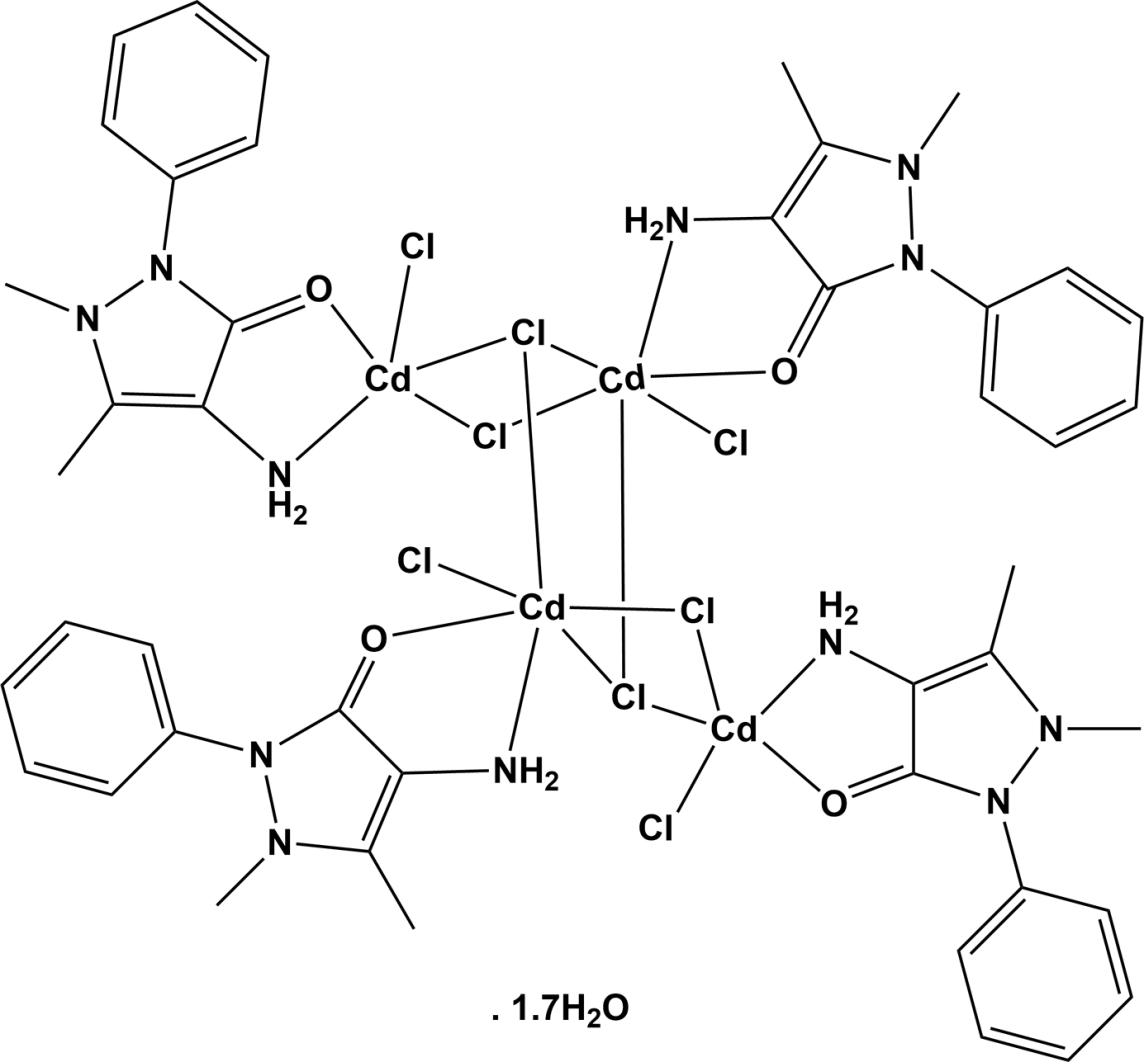


Cadmium(II) com­plexes in general are notable because of their excellent optical and electronic properties (Venkataramanan *et al.*, 1997 ▸). They also have uses in environmental and analytical chemistry, and materials science (Adhikari *et al.*, 2020 ▸; Roccanova *et al.*, 2017 ▸; Cheng *et al.*, 2017 ▸). A search of the Cambridge Structural Database (CSD, Version 5.46, up­date February 2025; Groom *et al.*, 2016 ▸) for transition-metal com­plexes of 4-AAP gave 13 hits. Two of these are cad­mium(II) com­plexes, *viz. catena*-[[(4-amino­anti­pyrine)aqua­(μ_2_-5-nitro­isophthalato)cadmium(II)] monohydrate] (CSD refcode CIXQIK; Wang *et al.*, 2008 ▸) and *catena*-[bis­(μ-4-amino-1,5-dimethyl-2-phenyl-1,2-di­hydro-3*H*-pyrazol-3-one)tetra­deca-μ-chloro-bis­(ethanol)hepta­cadmium(II)] (IQATAY; Hu *et al.*, 2012 ▸). In both of these, 4-amino­anti­pyrine acts as a bidentate ligand, donating the lone pairs of electrons from the amino N atom and the carbonyl O atom to the cadmium ion.

Depending on the stoichiometry and reaction conditions, the structure of the com­plex can vary. For example, when reacting 4-amino­anti­pyrine (0.0203 g, 0.1 mmol) with CdCl_2_·2.5H_2_O (0.142 g, 0.06 mmol) in a mixture of ethanol and ethyl ­acetate (1:3 *v*/*v*) at 343 K, Hu *et al.* (2012 ▸) synthesized a mono-periodic coordination polymer, *catena*-[bis­(μ-4-amino-1,5-dimethyl-2-phenyl-1,2-di­hydro-3*H*-pyrazol-3-one)tetra­deca-μ-chloro-bis­(ethanol)hepta­cadmium(II)]; structure IQATAY mentioned above. The com­plex unit possesses inversion symmetry and the asymmetric unit consists of 3.5 Cd^II^ atoms coordinated to seven bridging chloride ions, one bridging 4-amino­anti­pyrine ligand and one ethanol mol­ecule. In the present work, a new centrosymmetric tetra­nuclear cadmium(II) com­plex of 4-amino­anti­pyrine was synthesized when reacting an equimolar ratio of 4-AAP and CdCl_2_·2.5H_2_O using methanol as the solvent. Herein, we report on the structure and various properties of the title com­plex, [Cd_4_Cl_8_(C_11_H_13_N_3_O)_4_]·1.7H_2_O (**I**) (Scheme 1), and com­pare them to those of the hepta­cadmium com­plex IQATAY.

## 2. τ_6_, a sixfold geometry index

The mol­ecular structure of (**I**) was found to be centrosymmetric with two independent cadmium(II) atoms (see *Structural commentary* section). The two outer Cd^II^ atoms have fivefold coordination spheres, while the inner Cd^II^ atoms have sixfold coordination spheres. Previously, a number of authors have described methods to measure the size of distortions in polyhedra; some have included only distortions in the bond lengths from their average values (Robinson *et al.*, 1971 ▸; Muetterties & Guggenberger, 1974 ▸; Brown, 2006 ▸). Robinson *et al.* (1971 ▸) introduced the notion of quadratic elongation (QE) and this analysis is incorporated in *PLATON* (Spek, 2020 ▸). As of yet, no simple geometry index has been defined to describe the geometry of an octa­hedral coordination sphere. Deviations of the bond angles from their ideal values were largely ignored, but as shown below, when considered, they provide a simple method to calculate the various geometry indexes.

The fivefold geometry index τ_5_ was proposed by Addison *et al.* (1984 ▸) and is illustrated in Scheme 2 (τ_5_ = 0 for a perfect square pyramid and τ_5_ = 1 for a trigonal bipyramid). A fourfold geometry index τ_4_ was proposed by Yang *et al.* (2007 ▸) and is also illustrated in Scheme 2 (τ_4_ = 0 for a perfect square-planar geometry and τ_4_ = 1 for a pyramidal geometry). Following the reasoning of Yang *et al.* (2007 ▸), we propose a simple numerical estimation of the geometry of a sixfold coordination sphere; see Scheme 3 where τ_6_ = [(3 × 180°) – (α1 + α2 + α3)]/180° = 0 for a perfect octa­hedron where α1 = α2 = α3 = 180°. A value of 0.75 is obtained for a trigonal prismatic geometry, where α1 = α2 = α3 = 135° and a value of 1.00 is obtained for a penta­gonal pyramidal geometry, where α1 = α2 = α3 = α4 = α5 = 72°.

## 3. Structural commentary

**Figure 1:**
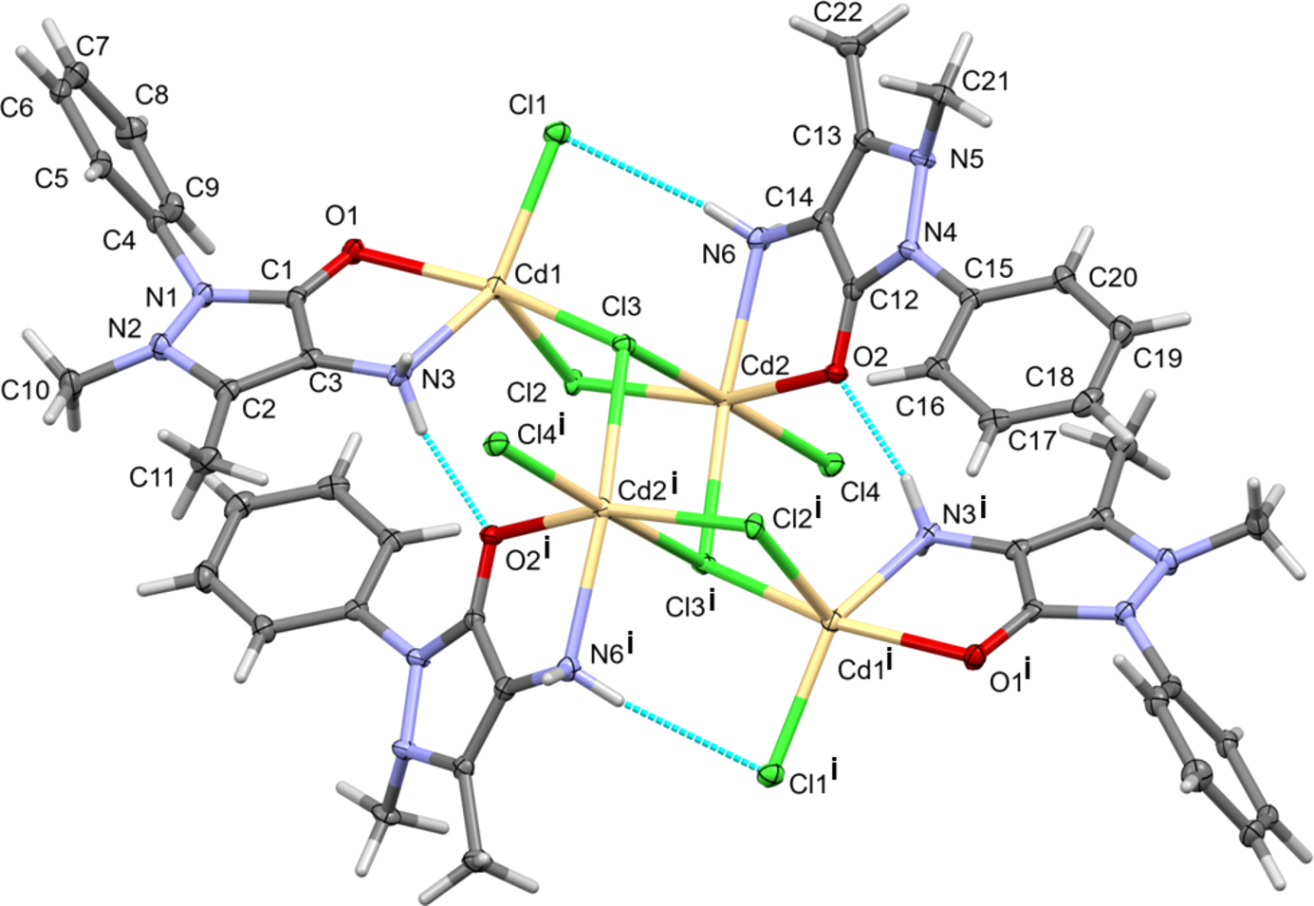
A view of the mol­ecular structure of (**I**), with displacement ellipsoids drawn at the 50% probability level. [Symmetry code: (i) −*x* + 2, −*y* + 2, −*z* + 1.]

The mol­ecular structure of (**I**) is illustrated in Fig. 1 ▸. This tetra­nuclear com­plex possesses inversion symmetry and crystallizes as a 1.7 hydrate. Selected bond lengths and angles in the com­plex are given in Table 1 ▸. The two independent Cd^II^ atoms of the asymmetric unit have different geometries, as shown in Figs. 1 ▸ and 2 ▸.

The outer Cd atoms [Cd1 and Cd1^i^; symmetry code: (i) −*x* + 2, −*y* + 2, −*z* + 1] have fivefold CdONCl_3_ coordination spheres with a distorted shape, and according to the definition of Addison *et al.* (1984 ▸), the structural index τ_5_ = (161.0° – 142.63°)/60° = 0.31. The inner Cd atoms (Cd2 and Cd2^i^) have sixfold CdONCl_4_ coordination spheres. The three principal (*trans*) bond angles O2—Cd2—Cl2, N6—Cd2—Cl3^i^ and Cl3—Cd2—Cl4 are 159.61 (4), 161.49 (4) and 170.34 (2)°, respectively, com­pared to 180° for a perfect octa­hedron. The τ_6_ geometry index gives a value of [540° – (159.61° + 161.49° + 170.34°)]/180° = 0.27, a significant distortion from the geometry of a perfect octa­hedron. The QE parameter for atom Cd2 is 1.027 (*PLATON*; Spek, 2020 ▸).
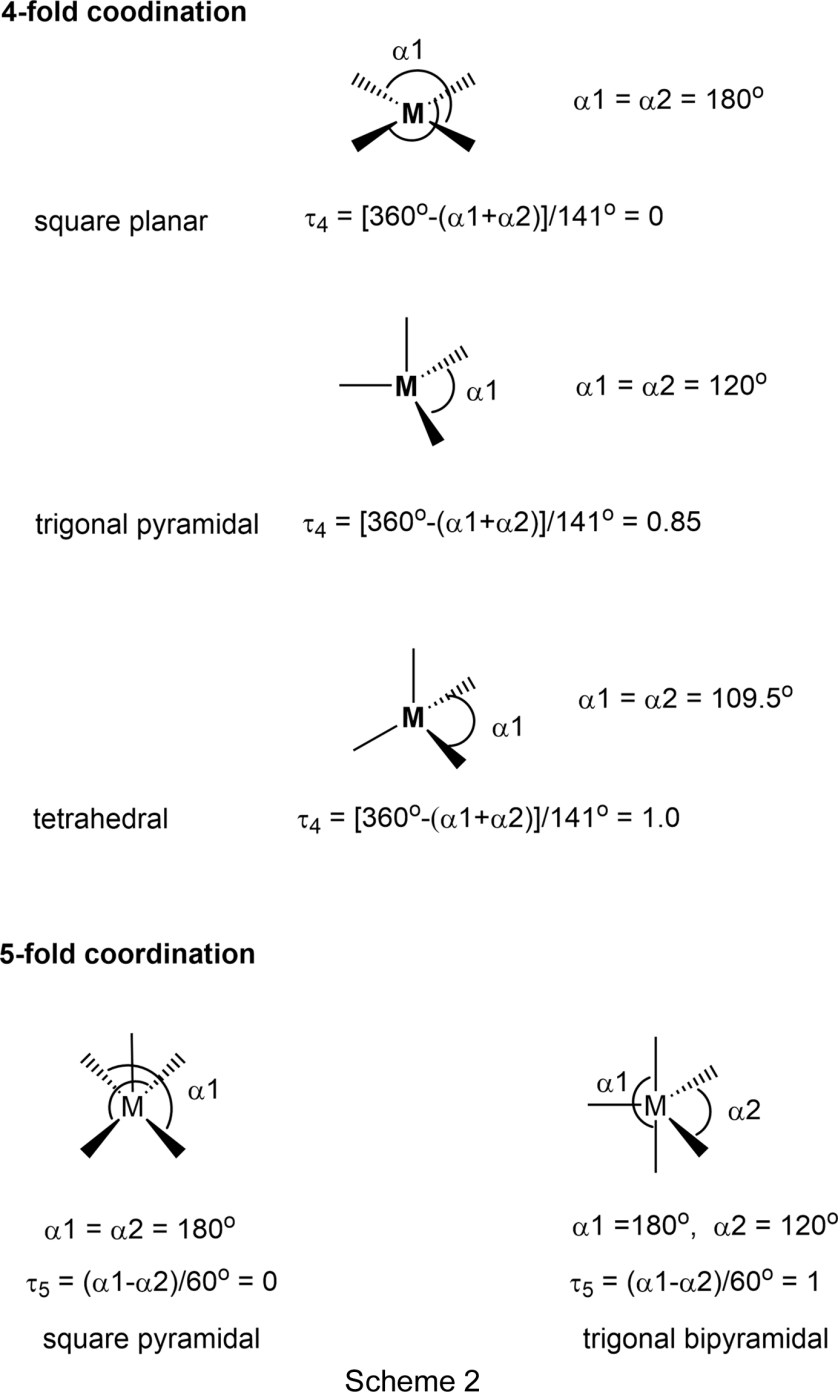


Comparing the coordination geometry of the four Cd atoms in IQATAY, it was found that the values of τ_6_ for atoms Cd1, Cd2, Cd3 and Cd4 are 0, 0.06, 0.16 and 0.17, respectively. Hence, in this case, atom Cd1, which lies on an inversion centre, has a perfect octa­hedral geometry. Atom Cd2 has a very small distortion from a perfect octa­hedron. Atoms Cd3 and Cd4 have distorted geometries but less so than that observed for atom Cd2 in com­plex (**I**). The QE parameters for atoms Cd1 to Cd4 in IQATAY are 1.002, 1.005, 1.013 and 1.018, respectively.
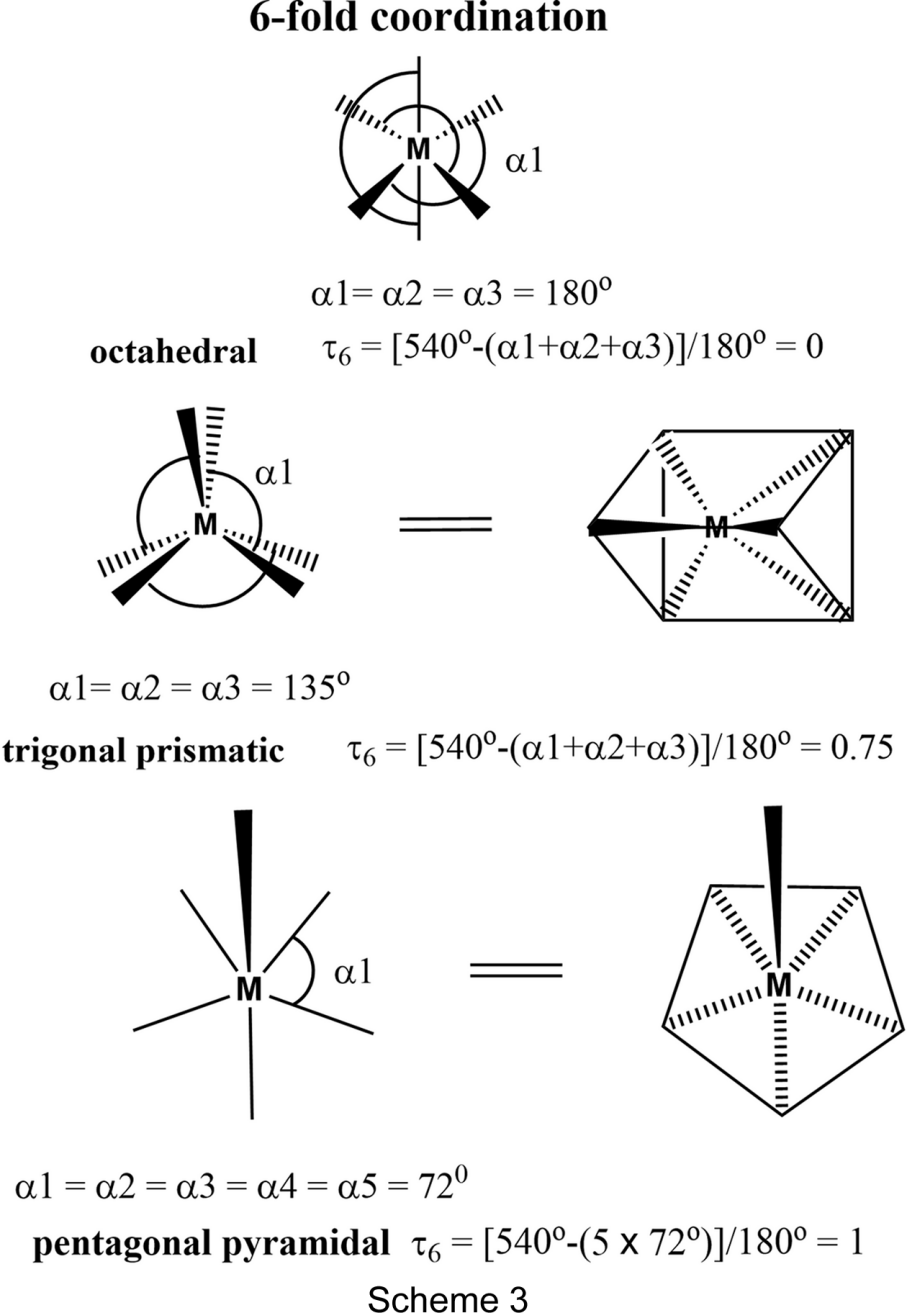


In (**I**), there are two terminal chlorido ligands (Cl1 and Cl4), one μ_2_-bridging Cl atom (Cl2) and one μ_3_-bridging Cl atom (Cl3); see Table 1 ▸. In IQATAY, there are three μ_2_-bridging Cl atoms and four μ_3_-bridging Cl atoms. The Cd—Cl bond lengths vary from 2.4486 (5) to 2.7631 (5) Å in (**I**), com­pared to a range of 2.551 (2)–2.779 (2) Å in IQATAY. The various Cd⋯Cd separations and Cd—Cl—Cd bridging angles in (**I**) are given in Table 1 ▸. These values are very similar to those observed for IQATAY.

The two independent 4-AAP mol­ecules in (**I**) have slightly different conformations. A view of the mol­ecular overlap of the two ligands [ligand 2 (involving atom O2) inverted over ligand 1 (involving atom O1)] is shown in Fig. S1 of the supporting information. In (**I**), the ligands are bidentate, coordinating to one Cd atom each time, while in IQATAY, the ligand bridges two Cd atoms. In (**I**), arene ring C4–C9 is inclined to the pyrazole ring mean plane (N1/N2/C1–C3; r.m.s. deviation = 0.05 Å) by 56.7 (1)°, while arene ring C15–C20 is inclined to the pyrazole ring mean plane (N4/N5/C12–C14; r.m.s. deviation 0.044 Å) by 45.2 (1)°. In IQATAY, the corresponding dihedral angle is larger at 62.1 (4)°. In (**I**), the pyrazole ring of ligand 1 (N1/N2/C1–C3) is almost parallel to arene ring C15^i^–C20^i^ of ligand 2, with a centroid–centroid separation of 3.749 (1) Å. The 4-AAP ligands in (**I**) coordinate to the Cd atoms *via* the carbonyl O atom and the 4-amino N atom. The Cd1—O1 bond length is 2.3498 (13) Å com­pared to 2.3107 (13) Å for Cd2—O2. In IQATAY, the corresponding Cd—O bond length is significantly shorter at 2.245 (6) Å. The Cd—N bond lengths in (**I**) are Cd1—N3 = 2.2863 (17) Å and Cd2—N6 = 2.4346 (17) Å, com­pared to 2.352 (7) Å for the corresponding bond in IQATAY.

Complex (**I**) is consolidated by intra­molecular N—H⋯Cl and N—H⋯O hy­dro­gen bonds, which are listed in Table 2 ▸ and illustrated in Fig. 1 ▸. The N6—H6N*A*⋯C1l hy­dro­gen bond involves the 4-amino group of ligand 2, while the N3—H3H*A*⋯O2^i^ hy­dro­gen bond involves the 4-amino group of ligand 1 and the carbonyl O atom of ligand 2.

## 4. Supra­molecular features

**Figure 3:**
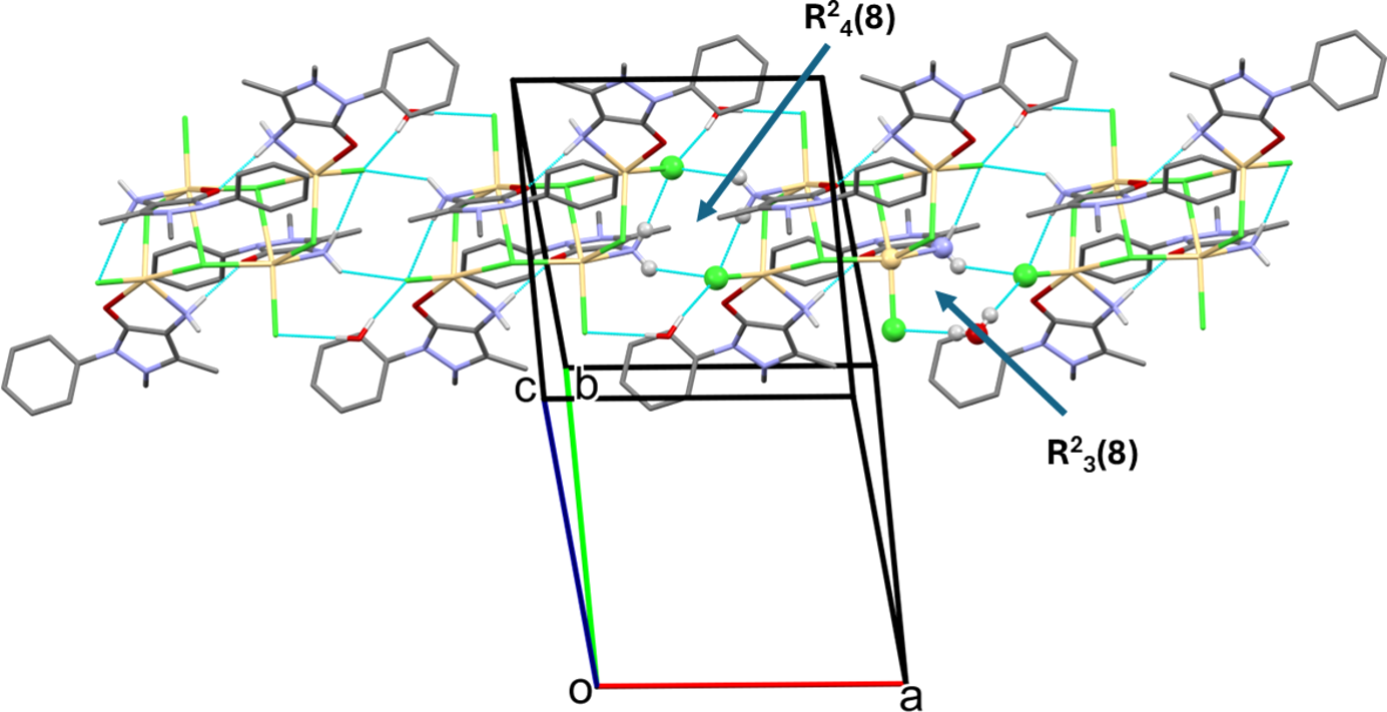
A partial view of the crystal packing of (**I**). Only the H atoms involved in the N—H⋯Cl and O—H⋯Cl hy­dro­gen bonds (Table 1 ▸) have been included.

**Figure 4:**
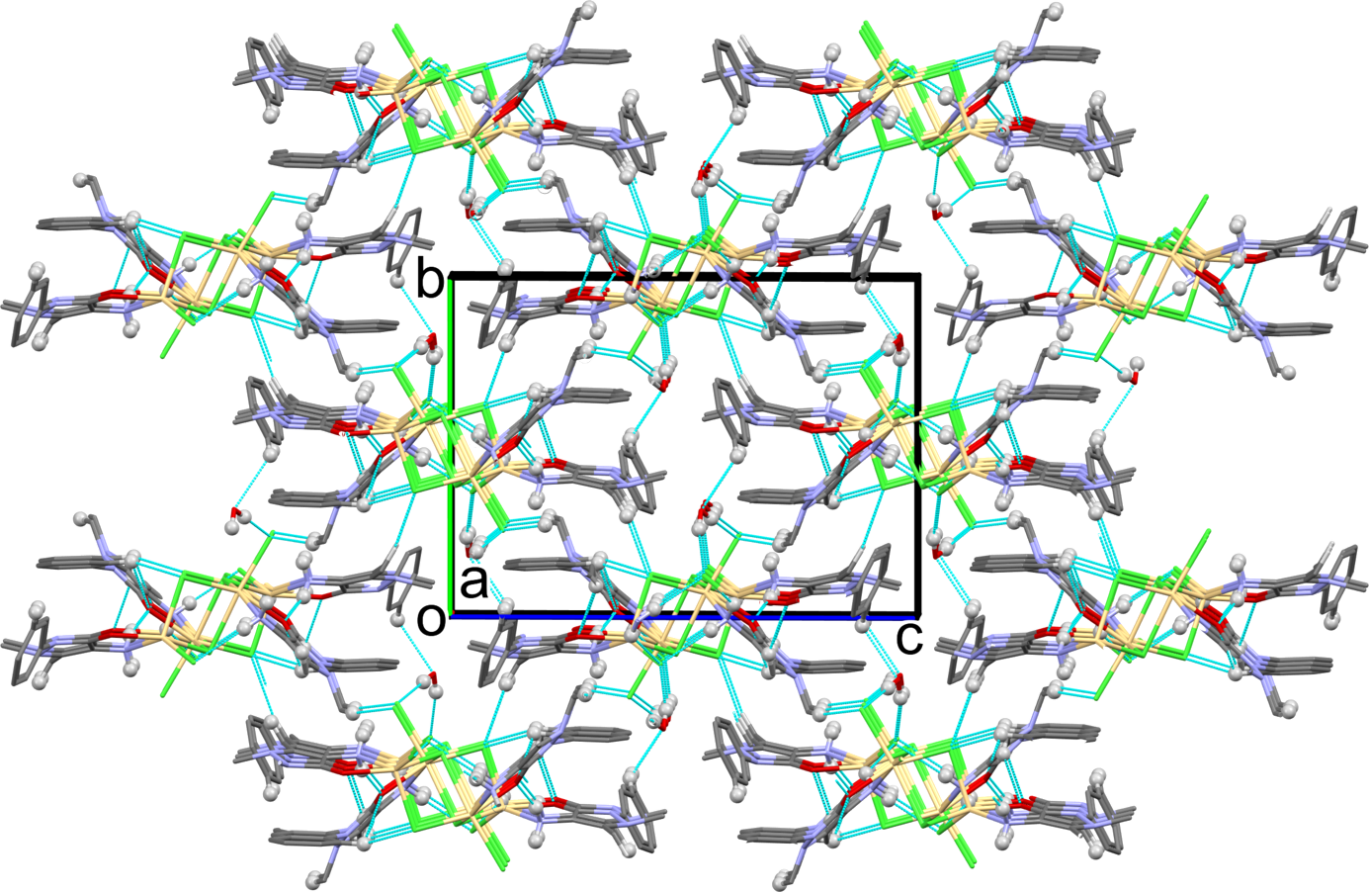
A view along the *a*-axis direction of the crystal packing of (**I**). Only the H atoms (grey spheres) involved in the various hy­dro­gen bonds (Table 1 ▸) have been included.

In the crystal of (**I**), the mol­ecules are linked by O—H⋯Cl hy­dro­gen bonds (Table 2 ▸), involving the partially occupied water mol­ecules (Cl⋯H—O—H⋯Cl). Together with N—H⋯Cl hy­dro­gen bonds (Table 2 ▸), chains are formed propagating along the *a*-axis direction (Fig. 3 ▸). The chains enclose two ring motifs. The first, 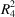
(8), involves the amine H atoms and chloride ion Cl1. The second, 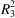
(8), involves an amine H atom, two chloride ions, the water H atoms and atom Cd1, as shown in Fig. 3 ▸. The chains are linked by a series of C—H⋯Cl and C—H⋯O hy­dro­gen bonds to form a three-dimensional structure (Fig. 4 ▸ and Table 2 ▸). Atom N3H*B* does not partake in hy­dro­gen bonding. This phenomenon is not unusual and has been observed previously (CSD; Groom *et al.*, 2016 ▸).

## 5. Hirshfeld surface analysis and fingerprint plots

**Figure 5:**
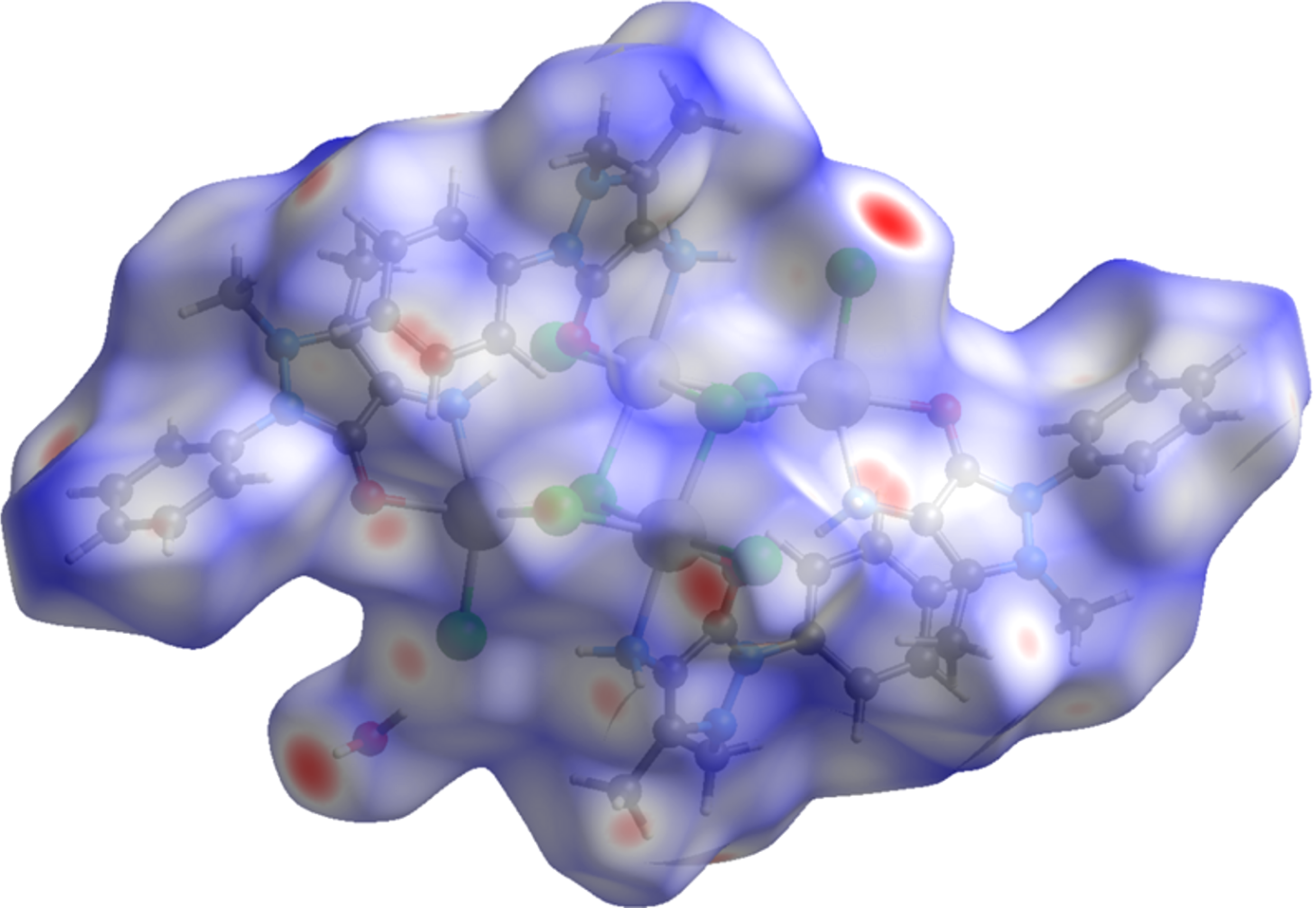
The Hirshfeld surface of (**I**) mapped over *d*_norm_

The Hirshfeld surface (HS) analysis and the associated two-dimensional fingerprint plots were generated with *CrystalExplorer17* (Spackman *et al.*, 2021 ▸) and inter­preted following the protocol of Tan *et al.* (2019 ▸). The Hirshfeld surface (HS) of (**I**) is illustrated in Fig. 5 ▸. It is colour-mapped with the normalized contact distance *d*_norm_ in the colour range from 0.00 to 1.41 a.u. There are a significant number of large red spots, indicating that in these regions the inter­atomic distances in the crystal are shorter than the sum of the van der Waals radii.

**Figure 6:**
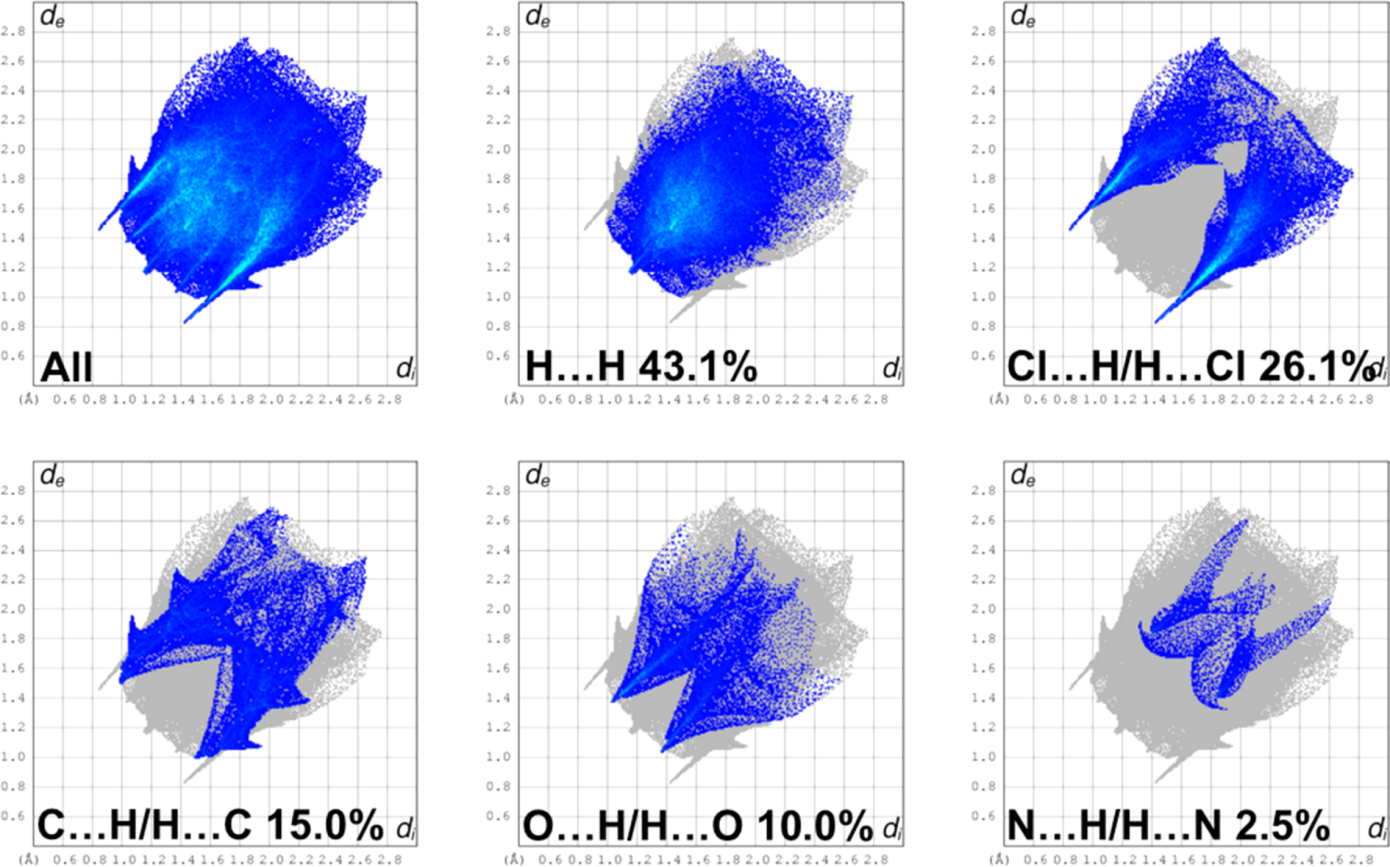
The full two-dimensional fingerprint plot for (**I**), and those delineated into H⋯H, Cl⋯H/H⋯Cl, C⋯H/H⋯C, O⋯H/H⋯O and N⋯H/H⋯N contacts.

The full two-dimensional fingerprint plot for (**I**), and those delineated into H⋯H, Cl⋯H/H⋯Cl, C⋯H/H⋯C, O⋯H/H⋯O and N⋯H/H⋯N contacts, are given in Fig. 6 ▸. The H⋯H contacts have a major contribution to the HS of 43.1%. The second most significant contributions are from the Cl⋯H/H⋯Cl contacts at 26.1%, with sharp peaks at *d*_i_ + *d*_e_ ≃ 2.2 Å. This reflects the presence of the seven H⋯Cl inter­actions in the crystal structure (see Table 2 ▸). The C⋯H/H⋯C inter­actions contribute 15.0%, with relatively sharp spikes at *d*_i_ + *d*_e_ ≃ 2.5 Å. The O⋯H/H⋯O inter­actions contribute 10%, with sharp peaks at *d*_i_ + *d*_e_ ≃ 2.35 Å. The sharp pincer-like peaks for these three inter­atomic inter­actions indicate that they are significant. Finally, the N⋯H/H⋯N contacts contribute 2.5%, while other inter­atomic contacts contribute less than 1% to the overall HS.

## 6. Database survey

A search of the Cambridge Structural Database (CSD, Version 5.46, update February 2025; Groom *et al.*, 2016 ▸) for sixfold-coordinated cadmium(II) metal com­plexes was carried out with the following restrictions: three-dimensional structure, *R* ≤ 0.05, no disorder, no ions, no polymers, single crystals only and no bidentate ligands with fewer than four atoms, such as nitrate or acetate. Over 720 hits were obtained.

**Figure 7:**
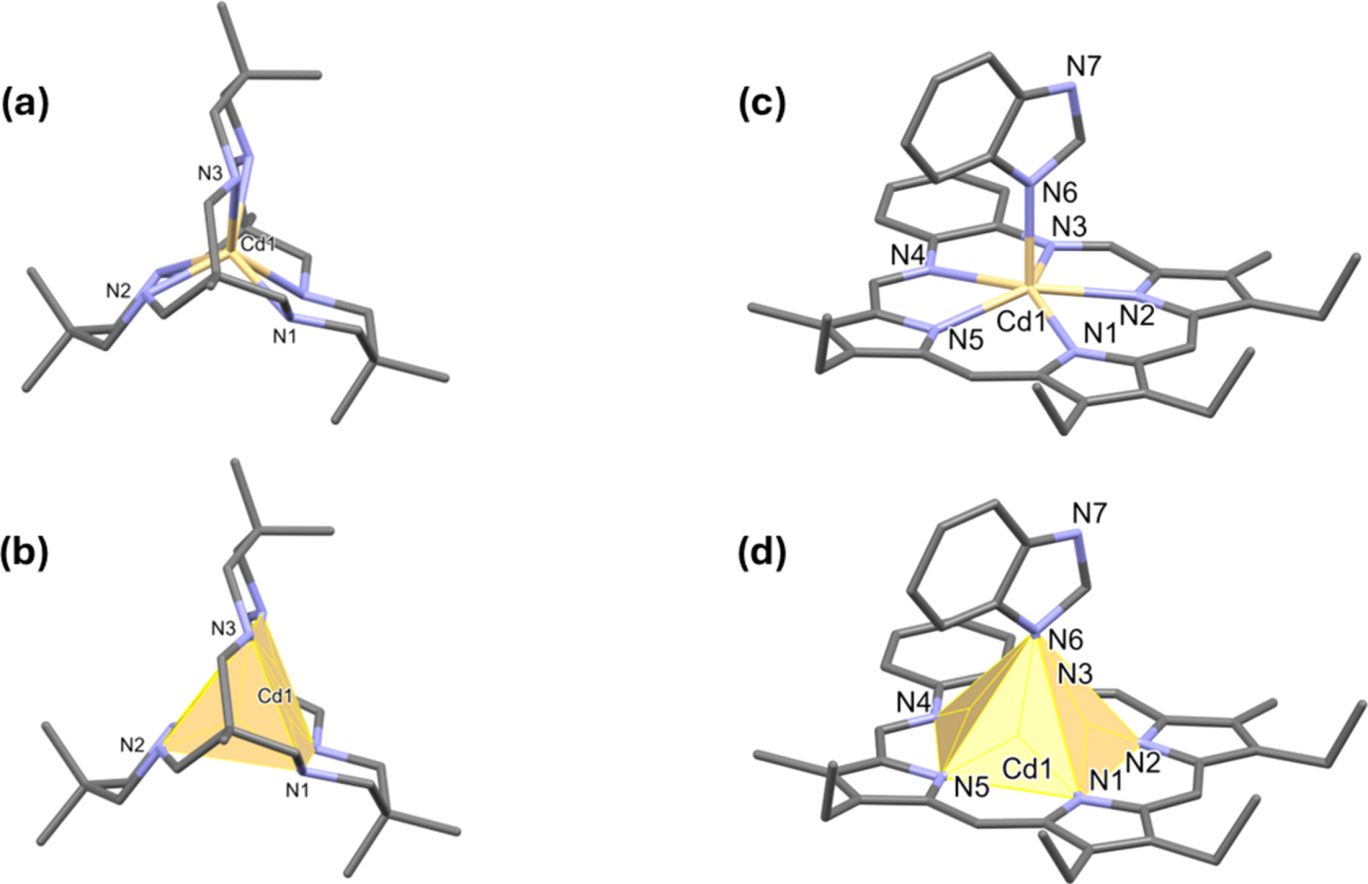
(*a*)/(*b*) The trigonal prismatic coordination sphere of ILUSAH (redrawn from Alcock *et al.*, 2016 ▸) and (*c*)/(*d*) the penta­gonal pyramidal coordination sphere of VAPSAG (redrawn from Sessler *et al.*, 1989 ▸).

Although the majority of the com­plexes have octa­hedral coordination spheres, there are a number of com­plexes with a trigonal prismatic geometry. One such com­plex is (1,5,5,9,13,13,20,20-octa­methyl-3,7,11,15,18,22-hexa­aza­bicyclo­[7.7.7]tricosa­ne)cadmium(II) dinitrate dihydrate (CSD refcode ULESAH; Alcock *et al.*, 2016 ▸), illustrated in Figs. 7 ▸(*a*) and 7(*b*). Here the structural index τ_6_ = 0.73 and the QE parameter for atom Cd1 is 1.24.

Examples of penta­gonal pyramidal geometry are rare. One example is (benzimidazole)(4,5,9,24-tetra­ethyl-10,23-dimethyl-13,20,25,26,27-pentaaza­penta­cyclo­[20.2.1.1^3,6^.1^8,11^.0^14,19^]hepta­cosa-1,3,5,7,9,11(27),12,14,16,18,20,22(25),23-trideca­ene)cad­mium(II) nitrate chloro­form solvate (VAPSAG; Sessler *et al.*, 1989 ▸), illustrated in Figs. 7 ▸(*c*) and 7(*d*); here the structural index τ_6_ = 1.02 (the QE parameter for atom Cd1 is 1.33). Another example is that of com­plex tetra­aqua­bis­(μ_2_-xanthurenato)dicadmium(II), with two Cd atoms related by a centre of symmetry (CIJBII; Tratar *et al.*, 2012 ▸). Here the structural index τ_6_ = 1.0 (QE parameter for atom Cd1 = 1.31).

## 7. Thermal analysis

To investigate the thermal characteristics of (**I**), a SQT-Q600 V20.9 Build 20 Universal Thermo Analytical system was used under a nitro­gen atmosphere and with a sample weight of 2.044 mg in an alumina crucible over a tem­per­a­ture range from 25 to 800 °C, with a heating rate of 20 °C per minute. The TGA and DTA results are illustrated in Fig. S2 of the supporting information.

The melting point of the com­plex, observed from the DTA curve, is 245 °C (518 K). According to the TGA curve, the first weight loss step (2.3%) corresponds to the loss of water mol­ecules present in the title com­plex. The second step (34.7% loss) is probably due to the gradual decom­position of one 4-amino­anti­pyrine mol­ecule, accom­panied by the release of chlorine gas. The third step (48.9% loss) may correspond to the loss of one 4-amino­anti­pyrine mol­ecule and one CdCl_2_ mol­ecule. The final step may be due to the degradation of the rest of the com­plex, leading to the formation of the final residue of CdO (observed 16.23%; calculated 16.63%)

## 8. Synthesis and spectroscopic data

An equimolar mixture of 4-amino­anti­pyrine (0.100 mmol) and cadmium(II) chloride (0.100 mmol) was dissolved in 20 ml methanol and refluxed at 363 K using an oil bath. After 6 h the solution was filtered and left aside at room tem­per­a­ture. Orange needle-like crystals of (**I**) were obtained by evaporation of the solvent over a period of one week. The melting point is 518 K (245 °C), as seen from the DTA curve (Fig. S2).

The FT–IR spectrum of (**I**) was recorded with a JASCO Infrared spectrometer (400–4000 cm^−1^) using the KBr pellet technique (Fig. S3 of the supporting information). The FT–IR spectrum of the free ligand (Swaminathan *et al.*, 2009 ▸) was com­pared with that of the cadmium com­plex to verify the coordination of 4-AAP with the Cd^2+^ ions. For 4-AAP, prominent peaks are seen at 3432 and 3325 cm^−1^, corresponding to the asymmetric and symmetric stretching modes of NH_2_, respectively, and at 1679 cm^−1^ for the carbonyl-group stretching band. In the FT–IR spectrum of (**I**), sharp peaks were observed around 3301, 3213 and 1631 cm^−1^. It may be seen that the vibrational frequencies of NH_2_ and C=O are shifted to lower frequencies upon Cd^2+^ coordination, consistent with the crystal structure.

## 9. Refinement details

Crystal data, data collection and structure refinement details are summarized in Table 3 ▸. The NH_2_ and water H atoms were located in difference Fourier maps and freely refined. C-bound H atoms were included in calculated positions and treated as riding, with C—H = 0.95–0.98 Å and *U*_iso_(H) = 1.2U_eq_(C) or 1.5*U*_eq_(methyl C).

## Supplementary Material

Crystal structure: contains datablock(s) I, global. DOI: 10.1107/S2056989025003123/hb8132sup1.cif

Structure factors: contains datablock(s) I. DOI: 10.1107/S2056989025003123/hb8132Isup2.hkl

Figs. S1-S4: Structural overlap of the two ligands; thermal analysis of com­pound I; FTIR spectrum of I; proton NMR spectrum of I. DOI: 10.1107/S2056989025003123/hb8132sup3.pdf

CCDC reference: 2441688

Additional supporting information:  crystallographic information; 3D view; checkCIF report

Additional supporting information:  crystallographic information; 3D view; checkCIF report

## Figures and Tables

**Figure 2:**
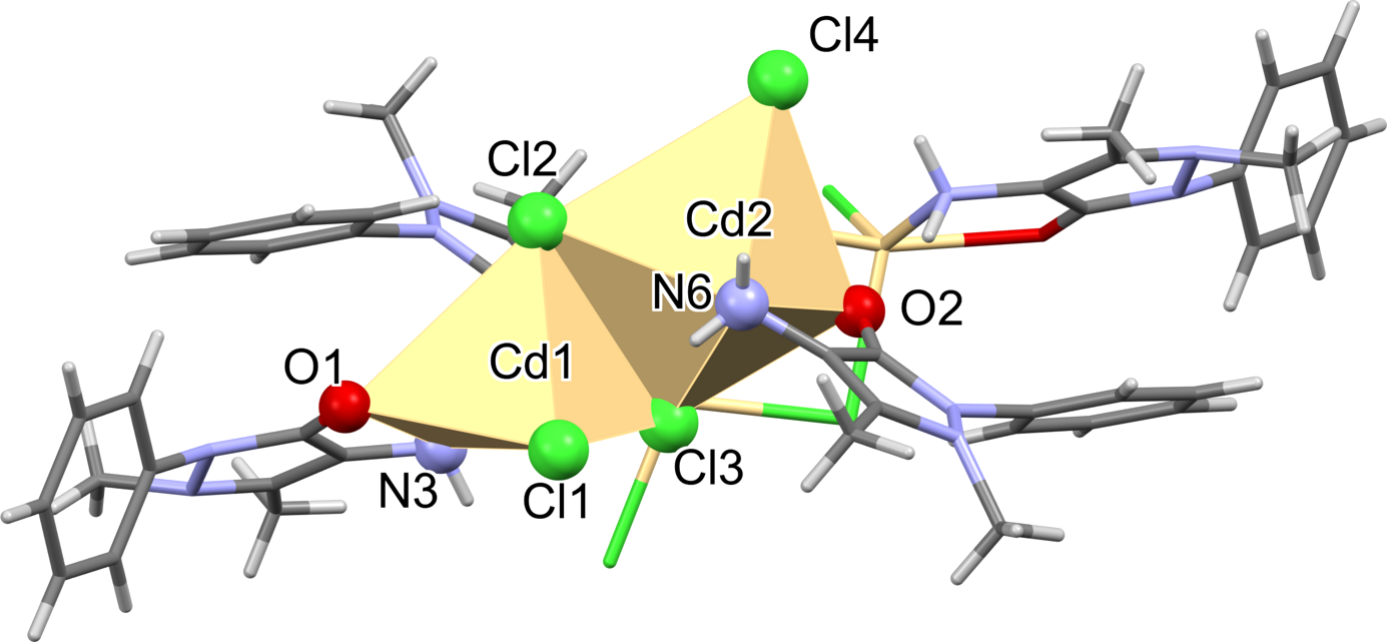
A partial view of (**I**), showing the different polyhedra for atoms Cd1 and Cd2 (*Mercury*; Macrae *et al.*, 2020 ▸).

**Table 1:** Selected geometric parameters (Å, °)

Cd1—O1	2.3498 (14)	Cd2—Cl2	2.5929 (5)
Cd1—N3	2.2863 (17)	Cd2—Cl3^i^	2.6734 (5)
Cd1—Cl1	2.4486 (5)	Cd2—Cl3	2.7631 (5)
Cd1—Cl2	2.5396 (5)	Cd2—Cl4	2.5157 (5)
Cd1—Cl3	2.6908 (5)	Cd1⋯Cd2	3.7831 (5)
Cd2—O2	2.3107 (13)	Cd2⋯Cd2^i^	4.0745 (4)
Cd2—N6	2.4346 (17)	Cd1⋯Cd2^i^	4.6003 (4)
			
N3—Cd1—Cl1	142.63 (5)	Cd1—Cl2—Cd2	94.958 (15)
O1—Cd1—Cl3	161.00 (4)	Cd2^i^—Cl3—Cd1	118.092 (16)
O2—Cd2—Cl2	159.61 (4)	Cd2^i^—Cl3—Cd2	97.069 (14)
N6—Cd2—Cl3^i^	161.49 (4)	Cd1—Cl3—Cd2	87.825 (14)
Cl4—Cd2—Cl3	170.343 (16)		

Symmetry code: (i) 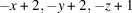.

**Table 2:** Hydrogen-bond geometry (Å, °)

*D*—H⋯*A*	*D*—H	H⋯*A*	*D*⋯*A*	*D*—H⋯*A*
N3—H3*NA*⋯O2^i^	0.88 (3)	1.89 (3)	2.747 (2)	166 (3)
N6—H6*NA*⋯Cl1	0.87 (3)	2.54 (3)	3.4073 (19)	172 (2)
N6—H6*NB*⋯Cl1^ii^	0.86 (3)	2.73 (3)	3.3718 (18)	133 (2)
O1*W*—H1*WA*⋯Cl4^ii^	0.85 (4)	2.45 (4)	3.292 (2)	176 (4)
O1*W*—H1*WB*⋯Cl1	0.89 (4)	2.34 (4)	3.220 (2)	168 (3)
C11—H11*A*⋯Cl2^iii^	0.98	2.82	3.732 (2)	156
C16—H16⋯Cl2^i^	0.95	2.82	3.589 (2)	138
C21—H21*C*⋯Cl4^iv^	0.98	2.69	3.647 (2)	165
C22—H22*C*⋯O1^ii^	0.98	2.51	3.417 (3)	154

Symmetry codes: (i) 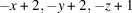; (ii) 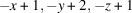; (iii) 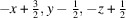; (iv) 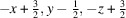.

**Table 3:** Experimental details

Crystal data	
Chemical formula	[Cd_4_Cl_8_(C_11_H_13_N_3_O)_4_]·1.7H_2_O
*M*_r_	1576.80
Crystal system, space group	Monoclinic, *P*2_1_/*n*
Temperature (K)	95
*a*, *b*, *c* (Å)	11.0702 (2), 13.5058 (2), 19.0700 (3)
β (°)	102.006 (2)
*V* (Å^3^)	2788.82 (8)
*Z*	2
Radiation type	Mo *K*α
μ (mm^−1^)	1.94
Crystal size (mm)	0.23 × 0.09 × 0.06

Data collection	
Diffractometer	Rigaku OD SuperNova Dual source diffractometer with an AtlasS2 detector
Absorption correction	Multi-scan (*CrysAlis PRO*; Rigaku OD, 2022 ▸)
*T*_min_, *T*_max_	0.659, 1.000
No. of measured, independent and observed [*I* > 2σ(*I*)] reflections	46639, 7273, 6505
*R*_int_	0.033
(sin θ/λ)_max_ (Å^−1^)	0.696

Refinement	
*R*[*F*^2^ > 2σ(*F*^2^)], *wR*(*F*^2^), *S*	0.022, 0.051, 1.05
No. of reflections	7273
No. of parameters	362
H-atom treatment	H atoms treated by a mixture of independent and constrained refinement
Δρ_max_, Δρ_min_ (e Å^−3^)	0.60, −0.50

Computer programs: *CrysAlis PRO* (Rigaku OD, 2022 ▸), *SUPERFLIP* (Palatinus & Chapuis, 2007 ▸), *JANA2006* (Petrıcěk *et al.*, 2023 ▸), *Mercury* (Macrae *et al.*, 2020 ▸), *SHELXL2019* (Sheldrick, 2015 ▸), *PLATON* (Spek, 2020 ▸) and *publCIF* (Westrip, 2010 ▸).

## supplementary crystallographic information

### Di-µ3-chlorido-1:2:3κ3Cl;2:3:4κ3Cl-di-µ2-chlorido-1:2κ2Cl;3:4κ2Cl-tetrakis[(4-amino-1,5-dimethyl-2-phenyl-2,3-dihydro-1H-pyrazol-3-one-κ2O,N4)chloridocadmium(II)] 1.7-hydrate Crystal data

**Table:**

[Cd_4_Cl_8_(C_11_H_13_N_3_O)_4_]·1.7H_2_O	*F*(000) = 1554
*M**_r_* = 1576.80	*D*_x_ = 1.878 Mg m^−^^3^
Monoclinic, *P*2_1_/*n*	Mo *K*α radiation, λ = 0.71073 Å
*a* = 11.0702 (2) Å	Cell parameters from 23338 reflections
*b* = 13.5058 (2) Å	θ = 2.4–29.4°
*c* = 19.0700 (3) Å	µ = 1.94 mm^−^^1^
β = 102.006 (2)°	*T* = 95 K
*V* = 2788.82 (8) Å^3^	Needle, orange
*Z* = 2	0.23 × 0.09 × 0.06 mm

### Di-µ3-chlorido-1:2:3κ3Cl;2:3:4κ3Cl-di-µ2-chlorido-1:2κ2Cl;3:4κ2Cl-tetrakis[(4-amino-1,5-dimethyl-2-phenyl-2,3-dihydro-1H-pyrazol-3-one-κ2O,N4)chloridocadmium(II)] 1.7-hydrate Data collection

**Table:**

Rigaku OD SuperNova Dual source diffractometer with an AtlasS2 detector	7273 independent reflections
Radiation source: micro-focus sealed X-ray tube	6505 reflections with *I* > 2σ(*I*)
Mirror monochromator	*R*_int_ = 0.033
Detector resolution: 5.2027 pixels mm^-1^	θ_max_ = 29.7°, θ_min_ = 2.2°
ω scans	*h* = −13→15
Absorption correction: multi-scan (CrysAlis PRO; Rigaku OD, 2022)	*k* = −17→17
*T*_min_ = 0.659, *T*_max_ = 1.000	*l* = −24→26
46639 measured reflections	

### Di-µ3-chlorido-1:2:3κ3Cl;2:3:4κ3Cl-di-µ2-chlorido-1:2κ2Cl;3:4κ2Cl-tetrakis[(4-amino-1,5-dimethyl-2-phenyl-2,3-dihydro-1H-pyrazol-3-one-κ2O,N4)chloridocadmium(II)] 1.7-hydrate Refinement

**Table:**

Refinement on *F*^2^	Primary atom site location: structure-invariant direct methods
Least-squares matrix: full	Secondary atom site location: difference Fourier map
*R*[*F*^2^ > 2σ(*F*^2^)] = 0.022	Hydrogen site location: mixed
*wR*(*F*^2^) = 0.051	H atoms treated by a mixture of independent and constrained refinement
*S* = 1.05	*w* = 1/[σ^2^(*F*_o_^2^) + (0.0206*P*)^2^ + 2.4714*P*] where *P* = (*F*_o_^2^ + 2*F*_c_^2^)/3
7273 reflections	(Δ/σ)_max_ = 0.004
362 parameters	Δρ_max_ = 0.60 e Å^−^^3^
0 restraints	Δρ_min_ = −0.50 e Å^−^^3^

### Di-µ3-chlorido-1:2:3κ3Cl;2:3:4κ3Cl-di-µ2-chlorido-1:2κ2Cl;3:4κ2Cl-tetrakis[(4-amino-1,5-dimethyl-2-phenyl-2,3-dihydro-1H-pyrazol-3-one-κ2O,N4)chloridocadmium(II)] 1.7-hydrate Special details

**Table:**

Geometry. All esds (except the esd in the dihedral angle between two l.s. planes) are estimated using the full covariance matrix. The cell esds are taken into account individually in the estimation of esds in distances, angles and torsion angles; correlations between esds in cell parameters are only used when they are defined by crystal symmetry. An approximate (isotropic) treatment of cell esds is used for estimating esds involving l.s. planes.

### Di-µ3-chlorido-1:2:3κ3Cl;2:3:4κ3Cl-di-µ2-chlorido-1:2κ2Cl;3:4κ2Cl-tetrakis[(4-amino-1,5-dimethyl-2-phenyl-2,3-dihydro-1H-pyrazol-3-one-κ2O,N4)chloridocadmium(II)] 1.7-hydrate Fractional atomic coordinates and isotropic or equivalent isotropic displacement parameters (Å^2^)

**Table:**

	*x*	*y*	*z*	*U*_iso_*/*U*_eq_	Occ. (<1)
Cd1	0.69699 (2)	0.94027 (2)	0.38615 (2)	0.01331 (4)	
Cd2	0.87944 (2)	1.07542 (2)	0.54768 (2)	0.01079 (4)	
Cl1	0.55065 (4)	0.86942 (3)	0.45253 (2)	0.01487 (9)	
Cl2	0.73018 (4)	1.11865 (3)	0.42784 (2)	0.01186 (9)	
Cl3	0.90013 (4)	0.89352 (3)	0.48490 (2)	0.01128 (9)	
Cl4	0.89700 (4)	1.23560 (3)	0.61639 (2)	0.01465 (9)	
O1	0.55409 (13)	0.94942 (10)	0.27639 (7)	0.0146 (3)	
O2	0.98088 (12)	0.98270 (10)	0.64391 (7)	0.0141 (3)	
N1	0.56201 (15)	0.92585 (12)	0.15576 (9)	0.0133 (3)	
N2	0.65230 (15)	0.89099 (13)	0.12006 (9)	0.0146 (3)	
N3	0.81571 (16)	0.90156 (13)	0.30520 (9)	0.0136 (3)	
H3NA	0.877 (3)	0.944 (2)	0.3144 (15)	0.034 (8)*	
H3NB	0.849 (2)	0.843 (2)	0.3157 (13)	0.024 (7)*	
N4	0.93489 (15)	0.86164 (12)	0.72079 (9)	0.0128 (3)	
N5	0.82718 (15)	0.82851 (12)	0.74095 (9)	0.0127 (3)	
N6	0.71297 (16)	0.99943 (13)	0.59441 (9)	0.0131 (3)	
H6NA	0.665 (3)	0.967 (2)	0.5601 (15)	0.029 (7)*	
H6NB	0.668 (3)	1.044 (2)	0.6087 (14)	0.026 (7)*	
C1	0.61290 (18)	0.93024 (13)	0.22807 (10)	0.0119 (4)	
C2	0.76124 (18)	0.88714 (14)	0.16873 (10)	0.0135 (4)	
C3	0.73903 (18)	0.90621 (14)	0.23532 (10)	0.0127 (4)	
C4	0.43370 (18)	0.91110 (14)	0.12652 (10)	0.0123 (4)	
C5	0.39217 (19)	0.82220 (14)	0.09338 (10)	0.0143 (4)	
H5	0.449080	0.771716	0.087636	0.017*	
C6	0.26638 (19)	0.80834 (15)	0.06883 (10)	0.0166 (4)	
H6	0.236818	0.748111	0.045759	0.020*	
C7	0.1838 (2)	0.88162 (16)	0.07774 (11)	0.0197 (4)	
H7	0.097618	0.871308	0.061310	0.024*	
C8	0.2263 (2)	0.97020 (16)	0.11061 (11)	0.0202 (4)	
H8	0.169172	1.020341	0.116638	0.024*	
C9	0.35225 (19)	0.98602 (15)	0.13480 (10)	0.0159 (4)	
H9	0.381885	1.047031	0.156594	0.019*	
C10	0.6400 (2)	0.91614 (17)	0.04415 (11)	0.0202 (4)	
H10A	0.561577	0.890208	0.016781	0.030*	
H10B	0.708564	0.886756	0.026115	0.030*	
H10C	0.641657	0.988261	0.038802	0.030*	
C11	0.87995 (19)	0.86561 (16)	0.14715 (12)	0.0186 (4)	
H11A	0.872718	0.803415	0.120124	0.028*	
H11B	0.945901	0.859609	0.190050	0.028*	
H11C	0.899560	0.919594	0.117035	0.028*	
C12	0.90388 (18)	0.93145 (13)	0.66823 (10)	0.0115 (4)	
C13	0.72966 (18)	0.86983 (14)	0.69393 (10)	0.0122 (4)	
C14	0.77348 (18)	0.93480 (14)	0.65018 (10)	0.0117 (4)	
C15	1.05447 (18)	0.85200 (13)	0.76565 (10)	0.0115 (4)	
C16	1.15402 (18)	0.84218 (14)	0.73232 (10)	0.0134 (4)	
H16	1.141132	0.837617	0.681590	0.016*	
C17	1.27289 (19)	0.83913 (14)	0.77424 (11)	0.0167 (4)	
H17	1.341938	0.834798	0.751990	0.020*	
C18	1.2910 (2)	0.84240 (15)	0.84841 (12)	0.0189 (4)	
H18	1.372259	0.839435	0.876910	0.023*	
C19	1.1906 (2)	0.84998 (16)	0.88080 (11)	0.0201 (4)	
H19	1.203312	0.851183	0.931614	0.024*	
C20	1.07113 (19)	0.85588 (14)	0.83977 (10)	0.0157 (4)	
H20	1.002367	0.862426	0.862051	0.019*	
C21	0.82669 (19)	0.72630 (14)	0.76782 (11)	0.0152 (4)	
H21A	0.905166	0.712708	0.801017	0.023*	
H21B	0.816074	0.679988	0.727439	0.023*	
H21C	0.758496	0.718190	0.792885	0.023*	
C22	0.60025 (18)	0.84102 (15)	0.69320 (11)	0.0172 (4)	
H22A	0.595252	0.811728	0.739527	0.026*	
H22B	0.572689	0.792542	0.654988	0.026*	
H22C	0.547234	0.899748	0.684607	0.026*	
O1W	0.3775 (2)	0.67988 (14)	0.46049 (11)	0.0262 (4)	0.85
H1WA	0.308 (4)	0.703 (3)	0.439 (2)	0.057 (13)*	0.85
H1WB	0.425 (4)	0.734 (3)	0.465 (2)	0.050 (11)*	0.85

### Di-µ3-chlorido-1:2:3κ3Cl;2:3:4κ3Cl-di-µ2-chlorido-1:2κ2Cl;3:4κ2Cl-tetrakis[(4-amino-1,5-dimethyl-2-phenyl-2,3-dihydro-1H-pyrazol-3-one-κ2O,N4)chloridocadmium(II)] 1.7-hydrate Atomic displacement parameters (Å^2^)

**Table:**

	*U*^11^	*U*^22^	*U*^33^	*U*^12^	*U*^13^	*U*^23^
Cd1	0.01297 (7)	0.01485 (7)	0.01124 (7)	−0.00084 (5)	0.00051 (5)	−0.00144 (5)
Cd2	0.01082 (7)	0.01089 (7)	0.01011 (7)	−0.00029 (5)	0.00088 (5)	0.00165 (5)
Cl1	0.0106 (2)	0.0169 (2)	0.0168 (2)	0.00061 (17)	0.00230 (17)	0.00041 (17)
Cl2	0.0138 (2)	0.0106 (2)	0.0098 (2)	0.00143 (17)	−0.00070 (16)	0.00113 (15)
Cl3	0.0095 (2)	0.0116 (2)	0.0125 (2)	−0.00016 (16)	0.00191 (16)	0.00141 (16)
Cl4	0.0149 (2)	0.0135 (2)	0.0168 (2)	−0.00281 (17)	0.00625 (18)	−0.00634 (17)
O1	0.0113 (7)	0.0193 (7)	0.0127 (7)	0.0002 (6)	0.0012 (5)	−0.0027 (5)
O2	0.0111 (7)	0.0178 (7)	0.0128 (7)	−0.0034 (6)	0.0009 (5)	0.0046 (5)
N1	0.0111 (8)	0.0164 (8)	0.0125 (8)	0.0003 (6)	0.0027 (6)	−0.0032 (6)
N2	0.0135 (8)	0.0186 (8)	0.0123 (8)	0.0022 (7)	0.0042 (6)	−0.0023 (6)
N3	0.0128 (8)	0.0116 (8)	0.0144 (8)	−0.0003 (7)	−0.0018 (7)	−0.0006 (6)
N4	0.0101 (8)	0.0154 (8)	0.0134 (8)	−0.0014 (6)	0.0033 (6)	0.0040 (6)
N5	0.0111 (8)	0.0134 (8)	0.0146 (8)	−0.0011 (6)	0.0050 (6)	0.0028 (6)
N6	0.0112 (8)	0.0135 (8)	0.0139 (8)	0.0014 (7)	0.0006 (7)	0.0017 (6)
C1	0.0132 (9)	0.0090 (9)	0.0127 (9)	−0.0021 (7)	0.0008 (7)	−0.0015 (7)
C2	0.0136 (9)	0.0101 (9)	0.0163 (9)	0.0001 (7)	0.0017 (8)	−0.0005 (7)
C3	0.0123 (9)	0.0086 (8)	0.0164 (10)	−0.0009 (7)	0.0013 (7)	−0.0004 (7)
C4	0.0125 (9)	0.0152 (9)	0.0089 (9)	−0.0003 (7)	0.0013 (7)	0.0005 (7)
C5	0.0181 (10)	0.0137 (9)	0.0103 (9)	0.0012 (8)	0.0013 (8)	0.0005 (7)
C6	0.0196 (10)	0.0187 (10)	0.0105 (9)	−0.0061 (8)	0.0005 (8)	−0.0006 (7)
C7	0.0147 (10)	0.0285 (11)	0.0149 (10)	−0.0024 (9)	0.0005 (8)	0.0005 (8)
C8	0.0163 (10)	0.0222 (11)	0.0217 (11)	0.0061 (9)	0.0034 (8)	0.0006 (8)
C9	0.0189 (10)	0.0138 (9)	0.0144 (9)	0.0023 (8)	0.0020 (8)	−0.0010 (7)
C10	0.0186 (11)	0.0301 (12)	0.0120 (10)	0.0017 (9)	0.0033 (8)	−0.0013 (8)
C11	0.0141 (10)	0.0195 (10)	0.0226 (11)	0.0018 (8)	0.0046 (8)	−0.0037 (8)
C12	0.0130 (9)	0.0116 (9)	0.0094 (9)	0.0002 (7)	0.0011 (7)	0.0000 (7)
C13	0.0120 (9)	0.0121 (9)	0.0124 (9)	0.0007 (7)	0.0024 (7)	−0.0016 (7)
C14	0.0108 (9)	0.0125 (9)	0.0114 (9)	0.0007 (7)	0.0011 (7)	−0.0016 (7)
C15	0.0113 (9)	0.0086 (8)	0.0138 (9)	0.0003 (7)	0.0007 (7)	0.0025 (7)
C16	0.0156 (10)	0.0103 (9)	0.0146 (9)	0.0006 (7)	0.0037 (8)	0.0011 (7)
C17	0.0136 (10)	0.0103 (9)	0.0267 (11)	0.0012 (8)	0.0051 (8)	0.0006 (8)
C18	0.0148 (10)	0.0133 (10)	0.0248 (11)	0.0008 (8)	−0.0044 (8)	0.0030 (8)
C19	0.0241 (11)	0.0197 (10)	0.0140 (10)	0.0000 (9)	−0.0014 (8)	0.0032 (8)
C20	0.0180 (10)	0.0159 (10)	0.0133 (9)	0.0014 (8)	0.0033 (8)	0.0022 (7)
C21	0.0173 (10)	0.0120 (9)	0.0172 (10)	−0.0002 (8)	0.0056 (8)	0.0046 (7)
C22	0.0122 (10)	0.0195 (10)	0.0205 (10)	−0.0018 (8)	0.0049 (8)	0.0020 (8)
O1W	0.0261 (11)	0.0187 (10)	0.0349 (11)	−0.0030 (9)	0.0087 (9)	0.0008 (8)

### Di-µ3-chlorido-1:2:3κ3Cl;2:3:4κ3Cl-di-µ2-chlorido-1:2κ2Cl;3:4κ2Cl-tetrakis[(4-amino-1,5-dimethyl-2-phenyl-2,3-dihydro-1H-pyrazol-3-one-κ2O,N4)chloridocadmium(II)] 1.7-hydrate Geometric parameters (Å, º)

**Table:**

Cd1—O1	2.3498 (14)	C5—C6	1.386 (3)
Cd1—N3	2.2863 (17)	C5—H5	0.9500
Cd1—Cl1	2.4486 (5)	C6—C7	1.382 (3)
Cd1—Cl2	2.5396 (5)	C6—H6	0.9500
Cd1—Cl3	2.6908 (5)	C7—C8	1.387 (3)
Cd2—O2	2.3107 (13)	C7—H7	0.9500
Cd2—N6	2.4346 (17)	C8—C9	1.391 (3)
Cd2—Cl2	2.5929 (5)	C8—H8	0.9500
Cd2—Cl3^i^	2.6734 (5)	C9—H9	0.9500
Cd2—Cl3	2.7631 (5)	C10—H10A	0.9800
Cd2—Cl4	2.5157 (5)	C10—H10B	0.9800
Cd1—Cd2	3.7831 (5)	C10—H10C	0.9800
Cd2—Cd2^i^	4.0745 (4)	C11—H11A	0.9800
Cd1—Cd2^i^	4.6003 (4)	C11—H11B	0.9800
O1—C1	1.261 (2)	C11—H11C	0.9800
O2—C12	1.259 (2)	C12—C14	1.413 (3)
N1—C1	1.378 (2)	C13—C14	1.367 (3)
N1—N2	1.403 (2)	C13—C22	1.482 (3)
N1—C4	1.428 (2)	C15—C20	1.388 (3)
N2—C2	1.361 (3)	C15—C16	1.388 (3)
N2—C10	1.465 (3)	C16—C17	1.391 (3)
N3—C3	1.425 (3)	C16—H16	0.9500
N3—H3NA	0.88 (3)	C17—C18	1.387 (3)
N3—H3NB	0.88 (3)	C17—H17	0.9500
N4—C12	1.367 (2)	C18—C19	1.383 (3)
N4—N5	1.400 (2)	C18—H18	0.9500
N4—C15	1.425 (2)	C19—C20	1.392 (3)
N5—C13	1.370 (2)	C19—H19	0.9500
N5—C21	1.473 (2)	C20—H20	0.9500
N6—C14	1.430 (2)	C21—H21A	0.9800
N6—H6NA	0.87 (3)	C21—H21B	0.9800
N6—H6NB	0.86 (3)	C21—H21C	0.9800
C1—C3	1.412 (3)	C22—H22A	0.9800
C2—C3	1.367 (3)	C22—H22B	0.9800
C2—C11	1.485 (3)	C22—H22C	0.9800
C4—C9	1.386 (3)	O1W—H1WA	0.85 (4)
C4—C5	1.390 (3)	O1W—H1WB	0.89 (4)
			
N3—Cd1—O1	77.62 (5)	C6—C5—H5	120.5
N3—Cd1—Cl1	142.63 (5)	C4—C5—H5	120.5
O1—Cd1—Cl1	94.93 (4)	C7—C6—C5	120.39 (19)
N3—Cd1—Cl2	111.23 (5)	C7—C6—H6	119.8
O1—Cd1—Cl2	104.82 (4)	C5—C6—H6	119.8
Cl1—Cd1—Cl2	106.087 (16)	C6—C7—C8	120.1 (2)
N3—Cd1—Cl3	84.54 (4)	C6—C7—H7	119.9
O1—Cd1—Cl3	161.00 (4)	C8—C7—H7	119.9
Cl1—Cd1—Cl3	95.335 (15)	C7—C8—C9	120.4 (2)
Cl2—Cd1—Cl3	87.599 (14)	C7—C8—H8	119.8
O2—Cd2—N6	76.15 (5)	C9—C8—H8	119.8
O2—Cd2—Cl4	94.60 (4)	C4—C9—C8	118.74 (19)
N6—Cd2—Cl4	98.87 (5)	C4—C9—H9	120.6
O2—Cd2—Cl2	159.61 (4)	C8—C9—H9	120.6
N6—Cd2—Cl2	91.46 (4)	N2—C10—H10A	109.5
Cl4—Cd2—Cl2	103.381 (16)	N2—C10—H10B	109.5
O2—Cd2—Cl3^i^	87.33 (4)	H10A—C10—H10B	109.5
N6—Cd2—Cl3^i^	161.49 (4)	N2—C10—H10C	109.5
Cl4—Cd2—Cl3	170.343 (16)	H10A—C10—H10C	109.5
Cl4—Cd2—Cl3^i^	90.613 (15)	H10B—C10—H10C	109.5
O2—Cd2—Cl3	77.99 (4)	C2—C11—H11A	109.5
N6—Cd2—Cl3	85.47 (4)	C2—C11—H11B	109.5
Cl2—Cd2—Cl3	85.035 (14)	H11A—C11—H11B	109.5
Cl3^i^—Cd2—Cl3	82.931 (14)	C2—C11—H11C	109.5
Cd1—Cl2—Cd2	94.958 (15)	H11A—C11—H11C	109.5
Cd2^i^—Cl3—Cd1	118.092 (16)	H11B—C11—H11C	109.5
Cd2^i^—Cl3—Cd2	97.069 (14)	O2—C12—N4	124.29 (18)
Cd1—Cl3—Cd2	87.825 (14)	O2—C12—C14	128.91 (18)
C1—O1—Cd1	106.61 (12)	N4—C12—C14	106.78 (16)
C12—O2—Cd2	109.64 (12)	C14—C13—N5	109.23 (17)
C1—N1—N2	108.41 (15)	C14—C13—C22	128.88 (18)
C1—N1—C4	124.28 (16)	N5—C13—C22	121.85 (17)
N2—N1—C4	121.00 (15)	C13—C14—C12	107.75 (17)
C2—N2—N1	107.55 (15)	C13—C14—N6	132.43 (18)
C2—N2—C10	124.72 (17)	C12—C14—N6	119.82 (17)
N1—N2—C10	118.20 (16)	C20—C15—C16	121.33 (18)
C3—N3—Cd1	107.89 (12)	C20—C15—N4	121.20 (17)
C3—N3—H3NA	116.3 (19)	C16—C15—N4	117.43 (17)
Cd1—N3—H3NA	104.2 (19)	C15—C16—C17	119.07 (18)
C3—N3—H3NB	113.2 (16)	C15—C16—H16	120.5
Cd1—N3—H3NB	108.9 (16)	C17—C16—H16	120.5
H3NA—N3—H3NB	106 (2)	C18—C17—C16	120.26 (19)
C12—N4—N5	108.87 (15)	C18—C17—H17	119.9
C12—N4—C15	124.31 (16)	C16—C17—H17	119.9
N5—N4—C15	122.94 (15)	C19—C18—C17	119.89 (19)
C13—N5—N4	106.86 (15)	C19—C18—H18	120.1
C13—N5—C21	123.36 (16)	C17—C18—H18	120.1
N4—N5—C21	117.39 (15)	C18—C19—C20	120.74 (19)
C14—N6—Cd2	104.86 (12)	C18—C19—H19	119.6
C14—N6—H6NA	111.8 (18)	C20—C19—H19	119.6
Cd2—N6—H6NA	109.1 (18)	C15—C20—C19	118.67 (19)
C14—N6—H6NB	113.7 (18)	C15—C20—H20	120.7
Cd2—N6—H6NB	110.6 (18)	C19—C20—H20	120.7
H6NA—N6—H6NB	107 (2)	N5—C21—H21A	109.5
O1—C1—N1	125.05 (18)	N5—C21—H21B	109.5
O1—C1—C3	128.77 (18)	H21A—C21—H21B	109.5
N1—C1—C3	106.15 (16)	N5—C21—H21C	109.5
N2—C2—C3	108.73 (17)	H21A—C21—H21C	109.5
N2—C2—C11	121.87 (17)	H21B—C21—H21C	109.5
C3—C2—C11	129.40 (19)	C13—C22—H22A	109.5
C2—C3—C1	108.52 (17)	C13—C22—H22B	109.5
C2—C3—N3	132.32 (18)	H22A—C22—H22B	109.5
C1—C3—N3	119.10 (17)	C13—C22—H22C	109.5
C9—C4—C5	121.40 (19)	H22A—C22—H22C	109.5
C9—C4—N1	118.13 (17)	H22B—C22—H22C	109.5
C5—C4—N1	120.42 (17)	H1WA—O1W—H1WB	101 (3)
C6—C5—C4	118.96 (19)		
			
C1—N1—N2—C2	−8.1 (2)	C6—C7—C8—C9	0.0 (3)
C4—N1—N2—C2	−161.26 (17)	C5—C4—C9—C8	1.4 (3)
C1—N1—N2—C10	−155.99 (17)	N1—C4—C9—C8	−175.99 (18)
C4—N1—N2—C10	50.8 (2)	C7—C8—C9—C4	−1.1 (3)
C12—N4—N5—C13	7.4 (2)	Cd2—O2—C12—N4	173.58 (15)
C15—N4—N5—C13	166.03 (17)	Cd2—O2—C12—C14	−8.5 (2)
C12—N4—N5—C21	150.92 (17)	N5—N4—C12—O2	172.46 (17)
C15—N4—N5—C21	−50.4 (2)	C15—N4—C12—O2	14.2 (3)
Cd1—O1—C1—N1	178.55 (15)	N5—N4—C12—C14	−5.9 (2)
Cd1—O1—C1—C3	0.8 (2)	C15—N4—C12—C14	−164.13 (17)
N2—N1—C1—O1	−172.91 (17)	N4—N5—C13—C14	−6.0 (2)
C4—N1—C1—O1	−20.8 (3)	C21—N5—C13—C14	−146.82 (17)
N2—N1—C1—C3	5.3 (2)	N4—N5—C13—C22	172.07 (17)
C4—N1—C1—C3	157.38 (17)	C21—N5—C13—C22	31.3 (3)
N1—N2—C2—C3	7.6 (2)	N5—C13—C14—C12	2.5 (2)
C10—N2—C2—C3	152.94 (19)	C22—C13—C14—C12	−175.43 (19)
N1—N2—C2—C11	−172.06 (17)	N5—C13—C14—N6	−177.43 (19)
C10—N2—C2—C11	−26.8 (3)	C22—C13—C14—N6	4.7 (4)
N2—C2—C3—C1	−4.4 (2)	O2—C12—C14—C13	−176.07 (19)
C11—C2—C3—C1	175.25 (19)	N4—C12—C14—C13	2.1 (2)
N2—C2—C3—N3	172.5 (2)	O2—C12—C14—N6	3.8 (3)
C11—C2—C3—N3	−7.8 (4)	N4—C12—C14—N6	−177.94 (16)
O1—C1—C3—C2	177.47 (19)	Cd2—N6—C14—C13	−177.09 (18)
N1—C1—C3—C2	−0.6 (2)	Cd2—N6—C14—C12	3.0 (2)
O1—C1—C3—N3	0.1 (3)	C12—N4—C15—C20	123.4 (2)
N1—C1—C3—N3	−178.00 (16)	N5—N4—C15—C20	−32.0 (3)
Cd1—N3—C3—C2	−177.59 (18)	C12—N4—C15—C16	−54.3 (3)
Cd1—N3—C3—C1	−0.9 (2)	N5—N4—C15—C16	150.31 (17)
C1—N1—C4—C9	68.2 (2)	C20—C15—C16—C17	−1.9 (3)
N2—N1—C4—C9	−142.99 (18)	N4—C15—C16—C17	175.82 (17)
C1—N1—C4—C5	−109.2 (2)	C15—C16—C17—C18	2.2 (3)
N2—N1—C4—C5	39.6 (3)	C16—C17—C18—C19	−0.8 (3)
C9—C4—C5—C6	−0.6 (3)	C17—C18—C19—C20	−1.0 (3)
N1—C4—C5—C6	176.71 (17)	C16—C15—C20—C19	0.2 (3)
C4—C5—C6—C7	−0.5 (3)	N4—C15—C20—C19	−177.46 (18)
C5—C6—C7—C8	0.8 (3)	C18—C19—C20—C15	1.3 (3)

Symmetry code: (i) −*x*+2, −*y*+2, −*z*+1.

### Di-µ3-chlorido-1:2:3κ3Cl;2:3:4κ3Cl-di-µ2-chlorido-1:2κ2Cl;3:4κ2Cl-tetrakis[(4-amino-1,5-dimethyl-2-phenyl-2,3-dihydro-1H-pyrazol-3-one-κ2O,N4)chloridocadmium(II)] 1.7-hydrate Hydrogen-bond geometry (Å, º)

**Table:**

*D*—H···*A*	*D*—H	H···*A*	*D*···*A*	*D*—H···*A*
N3—H3*NA*···O2^i^	0.88 (3)	1.89 (3)	2.747 (2)	166 (3)
N6—H6*NA*···Cl1	0.87 (3)	2.54 (3)	3.4073 (19)	172 (2)
N6—H6*NB*···Cl1^ii^	0.86 (3)	2.73 (3)	3.3718 (18)	133 (2)
O1*W*—H1*WA*···Cl4^ii^	0.85 (4)	2.45 (4)	3.292 (2)	176 (4)
O1*W*—H1*WB*···Cl1	0.89 (4)	2.34 (4)	3.220 (2)	168 (3)
C11—H11*A*···Cl2^iii^	0.98	2.82	3.732 (2)	156
C16—H16···Cl2^i^	0.95	2.82	3.589 (2)	138
C21—H21*C*···Cl4^iv^	0.98	2.69	3.647 (2)	165
C22—H22*C*···O1^ii^	0.98	2.51	3.417 (3)	154

Symmetry codes: (i) −*x*+2, −*y*+2, −*z*+1; (ii) −*x*+1, −*y*+2, −*z*+1; (iii) −*x*+3/2, *y*−1/2, −*z*+1/2; (iv) −*x*+3/2, *y*−1/2, −*z*+3/2.
