# Supplementary material for: Di-μ3-chlorido-1:2:3κ3Cl;2:3:4κ3Cl-di-μ2-chlorido-1:2κ2Cl;3:4κ2Cl-tetra­kis­[(4-amino-1,5-dimethyl-2-phenyl-2,3-di­hydro-1H-pyrazol-3-one-κ2N4,O)chlorido­cadmium(II)] 1.7-hydrate: a new six-coordinate geometry index, τ6
Source: Acta Crystallogr E Crystallogr Commun. 2025 Apr 11;81(Pt 5):393–400. doi: 10.1107/S2056989025003123 (PMC12054759; doi:10.1107/S2056989025003123)
Supplement: Supplementary file 3 [file e-81-00393-sup3.pdf]

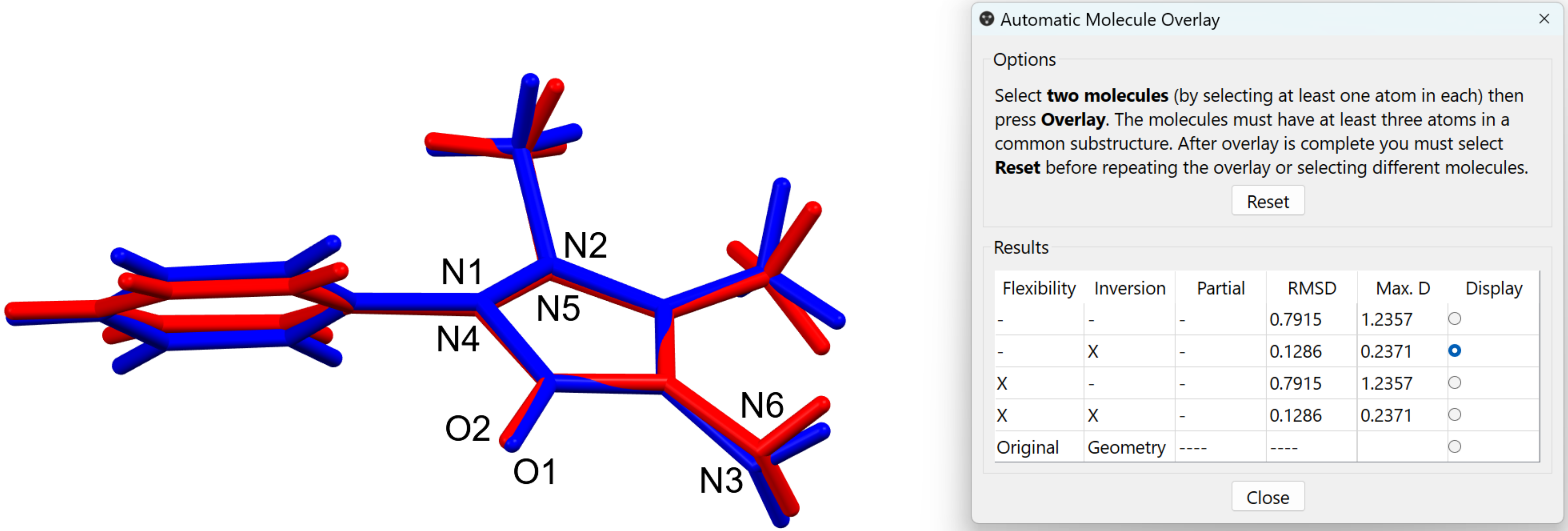

Flexibility	Inversion	Partial	RMSD	Max. D	Display
-	-	-	0.7915	1.2357	○
-	X	-	0.1286	0.2371	●
X	-	-	0.7915	1.2357	○
X	X	-	0.1286	0.2371	○
Original	Geometry	----	----		○

Fig. S1 Overlap of the two ligands (inverted ligand 1 on ligand 2) of complex I

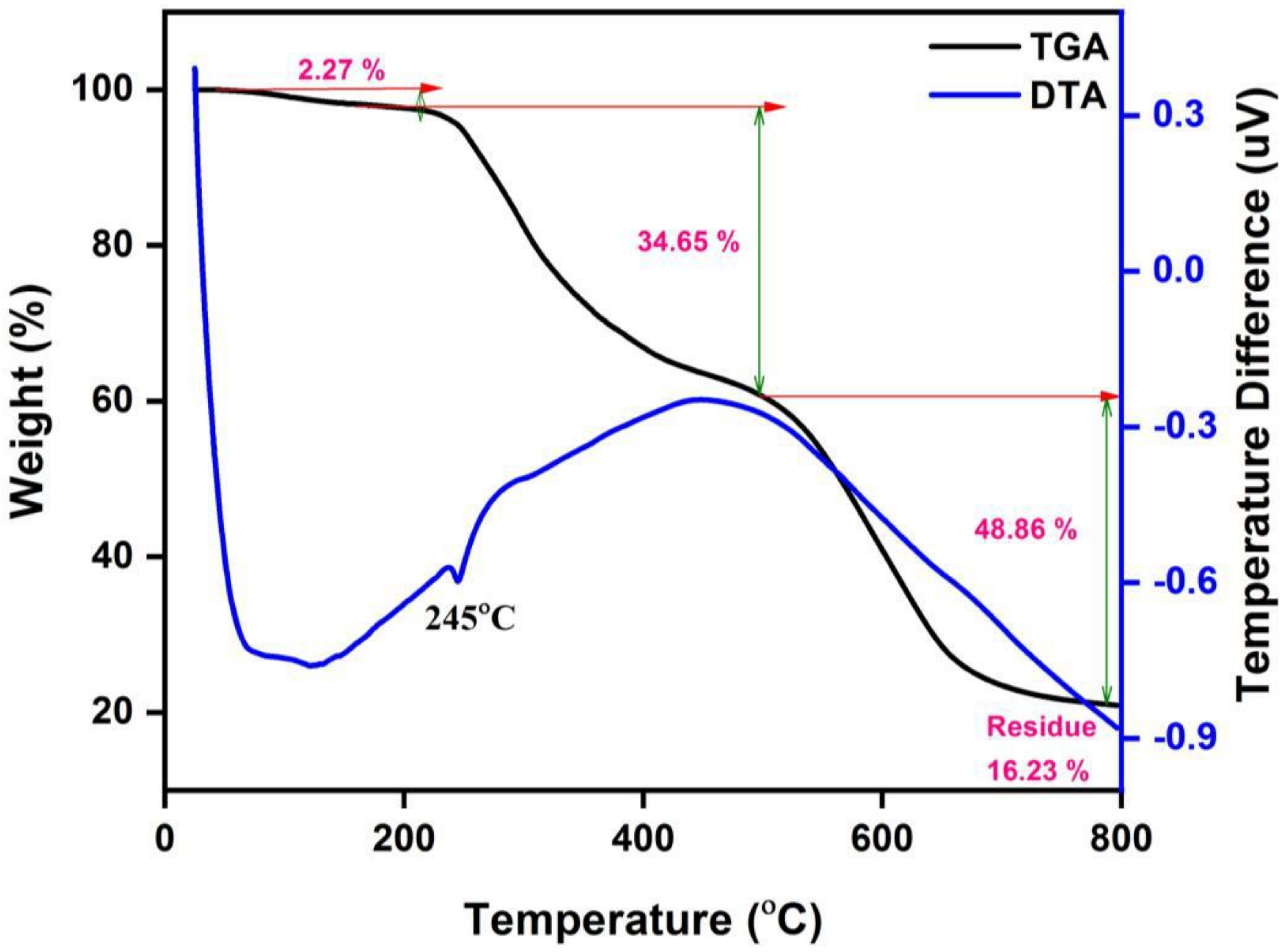

Fig. S2 Thermal analysis for compound I

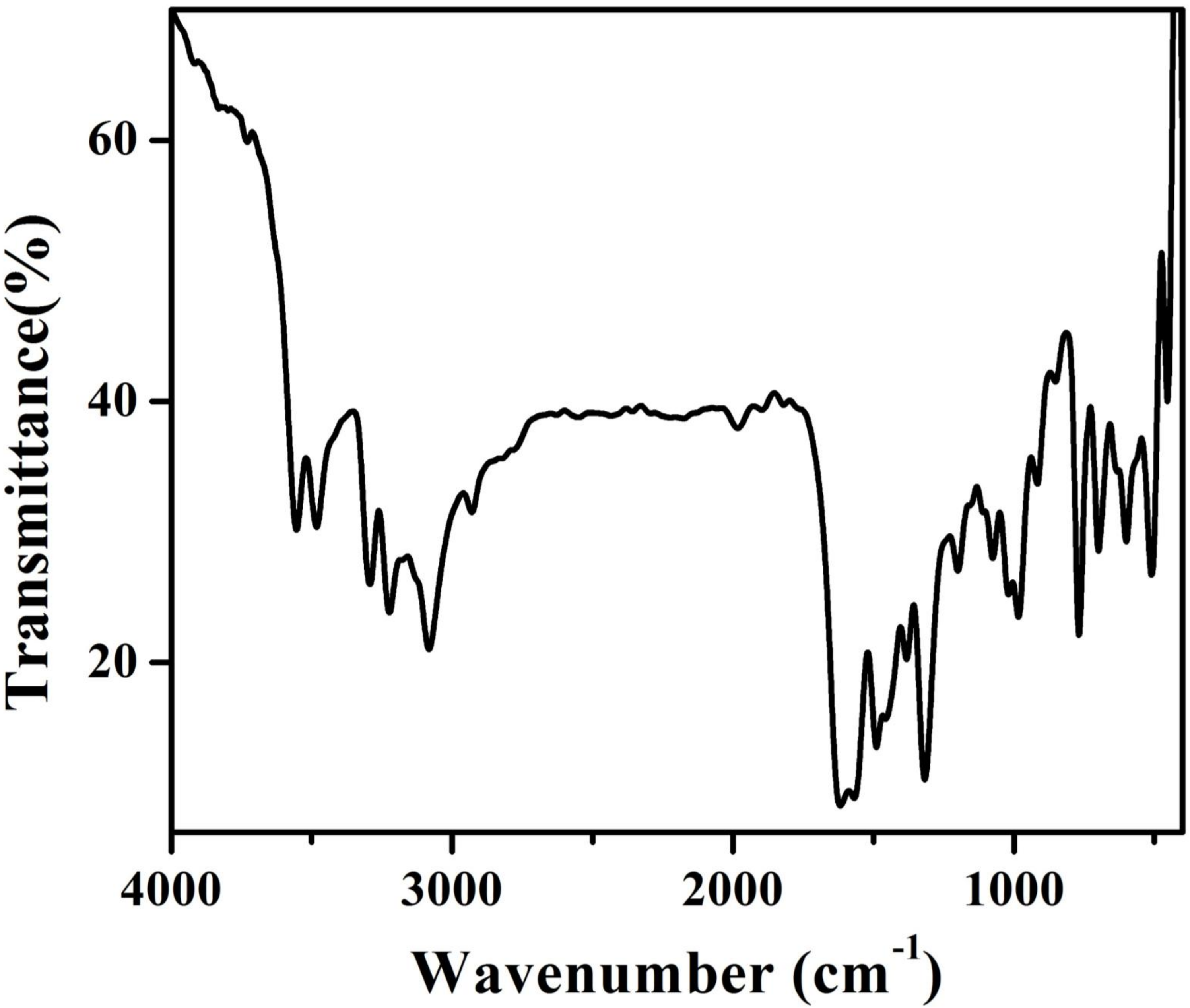

Fig. S3 FTIR spectrum for complex I

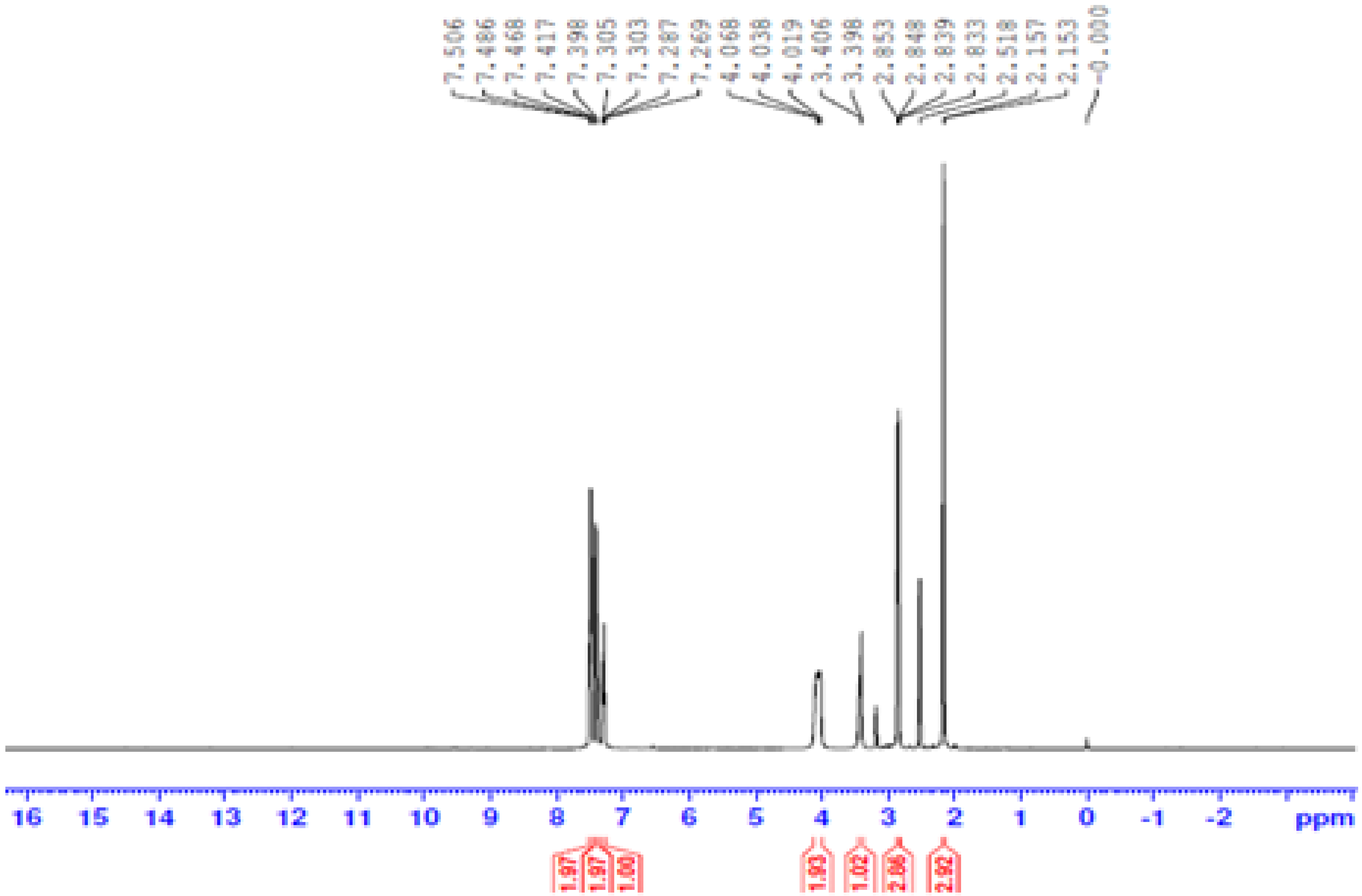

Fig. S4 ^{1}H NMR spectrum for compound I
